# LRRC15 expression indicates high level of stemness regulated by TWIST1 in mesenchymal stem cells

**DOI:** 10.1016/j.isci.2023.106946

**Published:** 2023-05-23

**Authors:** Kensuke Toriumi, Yuta Onodera, Toshiyuki Takehara, Tatsufumi Mori, Joe Hasei, Kanae Shigi, Natsumi Iwawaki, Toshifumi Ozaki, Masao Akagi, Mahito Nakanishi, Takeshi Teramura

**Affiliations:** 1 Department of Orthopedic Surgery, Kindai University Faculty of Medicine, Osaka-sayama, Osaka, Japan; 2 Institute of Advanced Clinical Medicine, Kindai University Hospital, Osaka-sayama, Osaka, Japan; 3 Life Science Institute, Kindai University, Osaka-sayama, Osaka, Japan; 4 Department of Orthopedic Surgery, Okayama University Faculty of Medicine, Okayama, Okayama, Japan; 5 Tokiwa Bio Inc, Tsukuba, Ibaraki, Japan

**Keywords:** Cell biology, Omics, Stem cells research, Transcriptomics

## Abstract

Mesenchymal stem cells (MSCs) are used as a major source for cell therapy, and its application is expanding in various diseases. On the other hand, reliable method to evaluate quality and therapeutic properties of MSC is limited. In this study, we focused on TWIST1 that is a transcription factor regulating stemness of MSCs and found that the transmembrane protein LRRC15 tightly correlated with the expression of TWIST1 and useful to expect TWIST1-regulated stemness of MSCs. The LRRC15-positive MSC populations in human and mouse bone marrow tissues highly expressed stemness-associated transcription factors and therapeutic cytokines, and showed better therapeutic effect in bleomycin-induced pulmonary fibrosis model mice. This study provides evidence for the important role of TWIST1 in the MSC stemness, and for the utility of the LRRC15 protein as a marker to estimate stem cell quality in MSCs before cell transplantation.

## Highlights

• TWIST1 regulates stemness in MSCs and its function is conserved in mice and human
• Transmembrane protein LRRC15 expression correlates with TWIST1 expression level
• LRRC15+ MSCs express stem cell markers and show therapeutic effect in BPF model mice
• LRRC15 is useful as a marker to evaluate quality of MSCs before cell transplantation

## Graphical abstract

**Figure:**
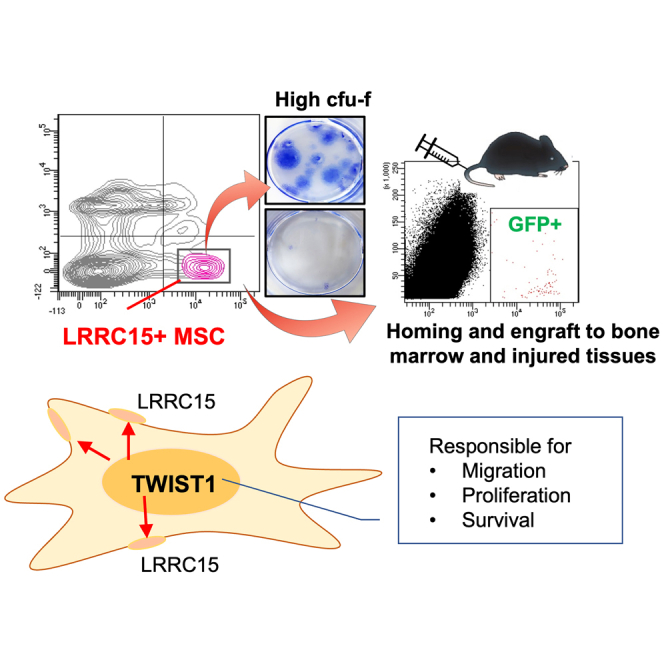

## Introduction

Mesenchymal stem cells (MSCs) are somatic stem cells present in various tissues including bone marrow, umbilical cord, and adipose tissue.^1^ Because of its multiple differentiation potentials and ability to produce therapeutic cytokines such as transforming growth factor β (TGFB), interleukin (IL)-10, and IL-1 receptor antagonist (IL1RA),^2^^,^^3^ MSCs are used in cell transplantation therapy for various diseases. On the other hand, method available to evaluate the MSC characteristics before undergoing cell transplantation is very limited.^4^ Reliable markers or method to qualify the MSCs are essential to improve safety and reproducibility of the MSC-based cell transplantation therapies. To date, CD146,^5^ CXCR4,^6^ and CD271^7^ have been reported as useful markers for human MSCs. However, the molecular mechanisms regulating expression of these markers are largely unknown, and it is also unclear which characters of MSCs are linked to the expression status of these markers. In mice, co-expression of Pdgfra and Sca1 has been reported as a useful marker to identify undifferentiated mouse MSCs with high level of stemness,^8^ but unfortunately, reproducibility and usefulness in human MSCs have not been well determined.

To identify the specific markers representing high level of stemness, we hypothesized that expression of such molecules should be tightly linked to the activity of key transcription factors regulating MSC stemness. Here, we focused on the basic-helix-loop-helix (bHLH) transcription factor Twist1 as the key molecule to establish MSC stemness. Twist1 regulates developmental processes such as lineage commitment and cellular differentiation in mesenchymal lineages,^9^^,^^10^ and also has been demonstrated to inhibit differentiation of MSCs.^11^ Additionally, it has been shown that expression level of Twist1 decreases dramatically during loss of stemness during *ex vivo* expansion.^12^ Boregowda et al. reported that TWIST1 can be a useful indicator of therapeutic properties by comparing expression of TWIST1 in the MSCs from multiple donors and undergoing TWIST1-overexpressing MSCs to bleomycin-induced injury models.^13^

In the present study, we determined importance of TWIST1 for the stem cell characteristics in human and mouse MSCs by overexpression and suppression of TWIST1. Furthermore, based on findings from the TWIST1-overexpressing MSCs, we identified the type I transmembrane protein 15-leucine-rich repeat-containing membrane protein (LRRC15) as a specific marker for live MSCs with higher proliferative and therapeutic potential *in vivo*.

## Results

### TWIST1 expression was diminished by cellular senescence in MSCs

**Figure 1:**
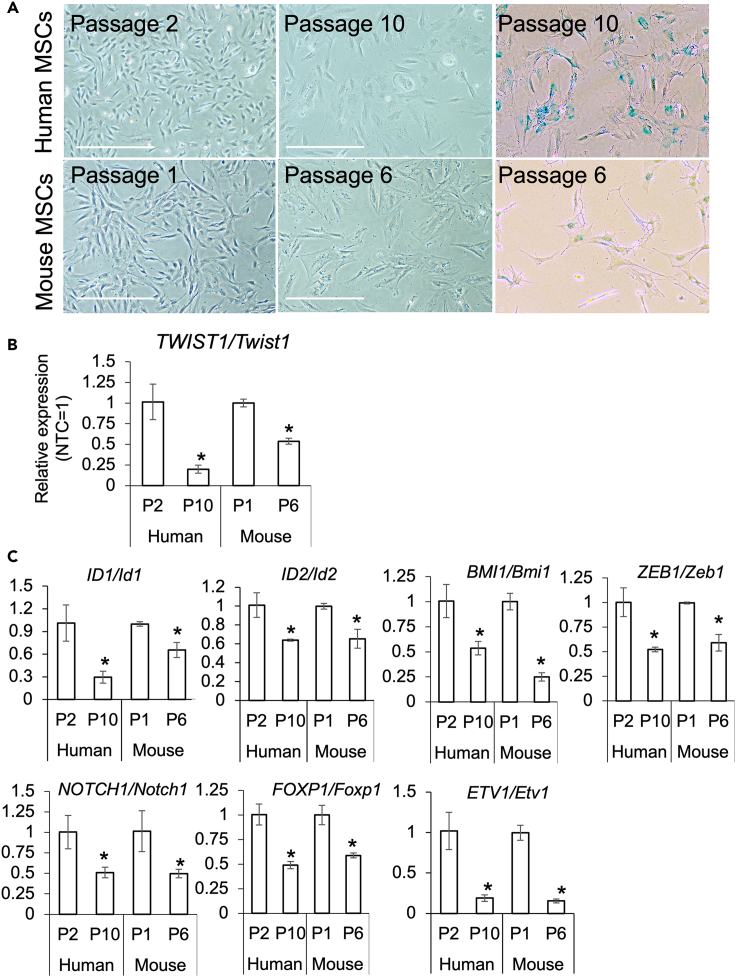
Senescence-associated changes of human and mice MSCs (A) Morphological changes of the MSCs by continuous cultures. Senescent cells showed β-galactosidase expressions. Scale bar = 500 μm. (B) *TWIST1/Twist1* expressions were suppressed in the senescent MSCs. Asterisk means significant difference detected at p < 0.05 (N = 3, biological replicates). (C) Gene expressions of stemness associated genes in the MSCs at each passage number. Asterisks mean significant difference were detected at p < 0.05 between control (N = 3, biological replicates).

MSCs undergo senescence through repeated *in vitro* passaging. By 10 times passages in human MSCs and 6 times passages in mice MSCs, the cells showed typical cellular senescence represented enlargement of cytoplasm and expression of β-galactosidase (Figures 1A and S1). In the senescent MSCs, *TWIST1* expression (Figure 1B) and stemness-associated transcription factors *Id1/ID1*, *Id2/ID2*, *Bmi1/BMI1*, *Zeb1/ZEB1*, *Notch1/NOTCH1*, *Foxp1/FOXP1*, and *Etv1/ETV1* were significantly suppressed in both mouse and human MSCs (Figure 1C).

### Suppression of TWIST1 attenuated stemness of the MSCs

**Figure 2:**
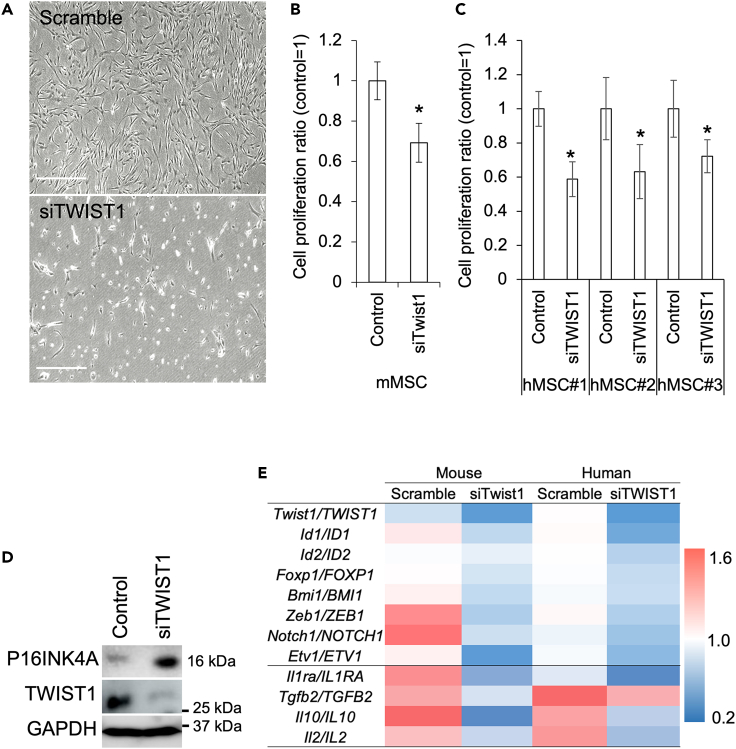
Suppression of TWIST1/Twist1 affected stem cell characteristics of the MSCs (A) Morphological changes of the MSCs by siTWIST1 treatment. Scale bar = 500 μm. (B) Relative cell numbers counted at 72 h after siRNA treatment in the mouse primary MSCs (mMSCs). Asterisks mean significant difference were detected at p < 0.05 between control (N = 3, biological replicates). (C) Relative cell numbers counted 72 h after siRNA treatment in human MSCs (hMSCs). Asterisks mean significant difference were detected at p < 0.05 between control (N = 3, biological replicates). (D) Western blot analysis of a cell cycle suppression and senescence marker P16INK4A in the siTWIST1-treated MSCs. (E) Heatmap comparisons of stemness-associated gene expressions between the scrambled RNA-transfected control and the siTWIST1/Twist1-treated MSCs both in human and mice. Numbers on the color scale are relative values to the no-treatment control as 1.0 and the values represent the average of three independent samples.

To examine the role of TWIST1 in the stem cell maintenance, we treated mouse and human MSCs with siRNA against TWIST1 (siTIWST1). In the siTWIST1-treated MSCs, cell proliferation was reduced to 70% in mice and approximately 60% in humans of the control MSCs treated with scrambled RNA (Figures 2A–2C). When the siTWIST1-treated human MSCs were examined by time-lapse microscopy, cell motility was also dramatically suppressed (Videos S1 and S2). Western blot analysis showed that P16INK4A expression was significantly upregulated in the siTWIST1-treated MSCs (Figure 2D). qRT-PCR showed that gene expression of the stemness-associated transcription factors and therapeutic cytokines were downregulated in the siTWIST1-treated MSCs (Figure 2E).

Video S1. Changes in proliferation and migration of MSCs after control siRNA treatment, related to Figure 2 The movie shows time-lapse imaging of the MSCs treated with scrambled RNA as control experiment. This movie file shows 24 h observation in fast forward.

Video S2. Changes in proliferation and migration of MSCs after siTWIST1 treatment, related to Figure 2 The movie shows time-lapse imaging of the MSCs treated with siTWIST1. This movie file shows 24 h observation in fast forward.

### Overexpression of TWIST1 enhances proliferation and gene expression of stemness-associated transcription factors in human and mouse MSCs

**Figure 3:**
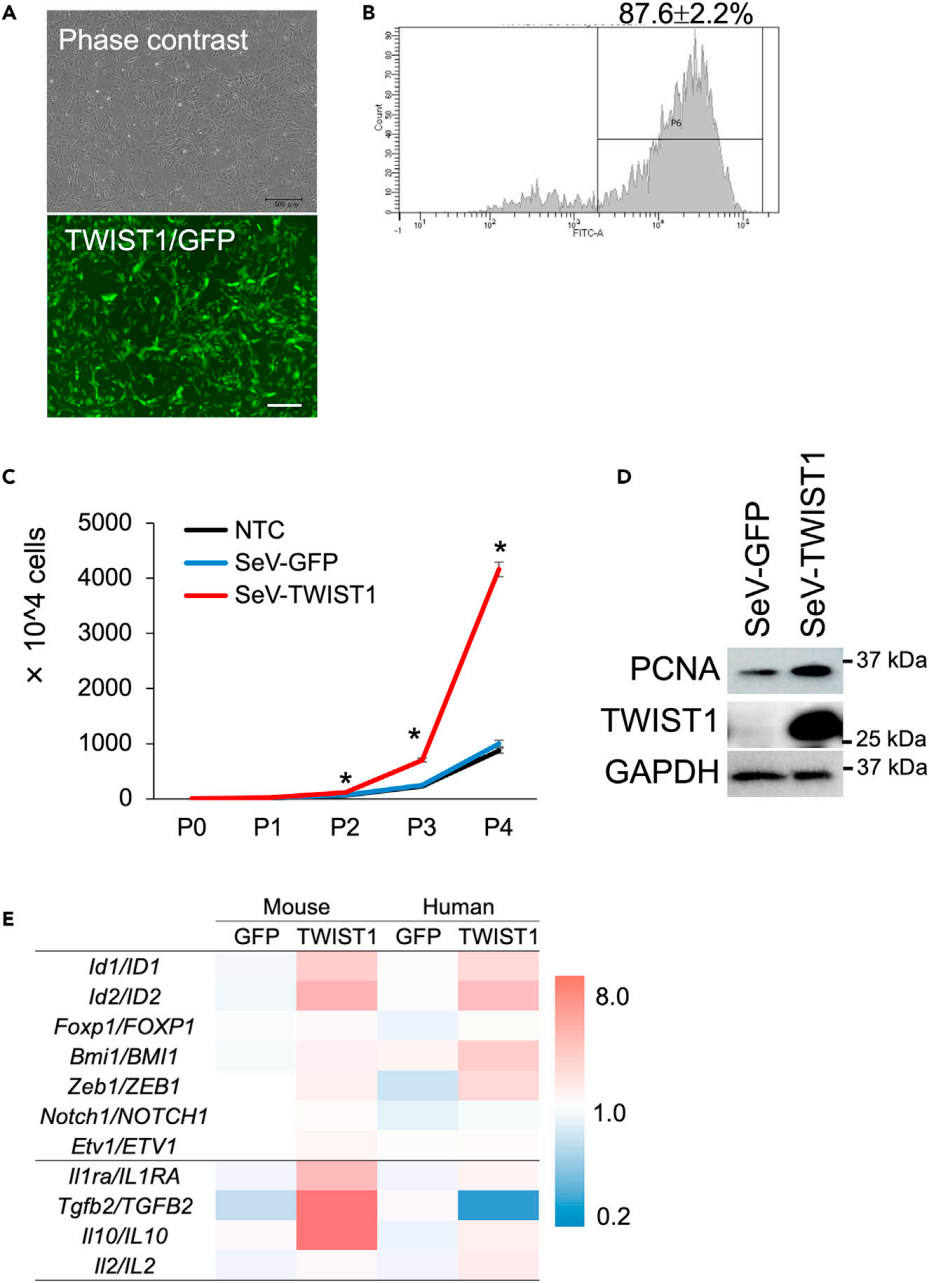
Overexpression of *TWIST1/Twist1* by a Sendai virus vector (SeV) in mouse and human MSCs (A) SeV resulted in high-efficient and stable gene introduction both in human and mice MSCs. Scale bar = 500 μm. (B) Efficiency of the SeV-TWIST1 transfection was observed by FACS analysis based on the GFP expression that was co-expressed with TWIST1. (C) TWIST1-overexpresing MSCs showed higher proliferation activity than the control cells transfected with SeV-GFP. Asterisks mean significant difference were detected at p < 0.05 (N = 3, biological replicates). (D) Western blot analysis of a proliferating cell marker PCNA in the TWIST1-overexpressing MSCs. (E) Gene expressions of the stemness-associated transcription factors and therapeutic cytokines in the SeV-GFP (control) and SeV-TWIST1-treated MSCs. Numbers on the color scale are relative values. Expression values used for the heatmap were the average obtained in three independent samples.

To observe effect of TWIST1 overexpression in the MSCs, we used a Sendai virus (SeV) system that enables high-efficient and stable expression of TWIST1. Transfection of the SeV cording TWIST1 gene (SeV-TWIST1) resulted in over 80% transfection efficiency without drug screening (Figures 3A and 3B), and by subsequent puromycin screening for 48 h, 100% of the cells expressed the exogenous TWIST1. MSCs transfected with the SeV-TWIST1 showed significant enhancement in cell proliferation (Figures 3C and S2). Western blot analysis showed that cell proliferation marker protein proliferating cell nuclear antigen (PCNA) as upregulated by the TWIST1 overexpression (Figure 3D). In the TWIST1-overexpressing MSCs, stemness-associated transcription factors *Id1/ID1*, *Id2/ID2*, *Bmi1/BMI1*, and *Zeb1/ZEB1*, and the therapeutic property-related cytokines *Il1ra/IL1Ra* and *Il10/IL10* were upregulated compared with the SeV-GFP-transfected control MSCs in both mice and humans (Figure 3E).

### Expression of cell surface protein LRRC15 correlates with the expression level of TWIST1 and can be used as a marker for TWIST1-mediated stemness

**Figure 4:**
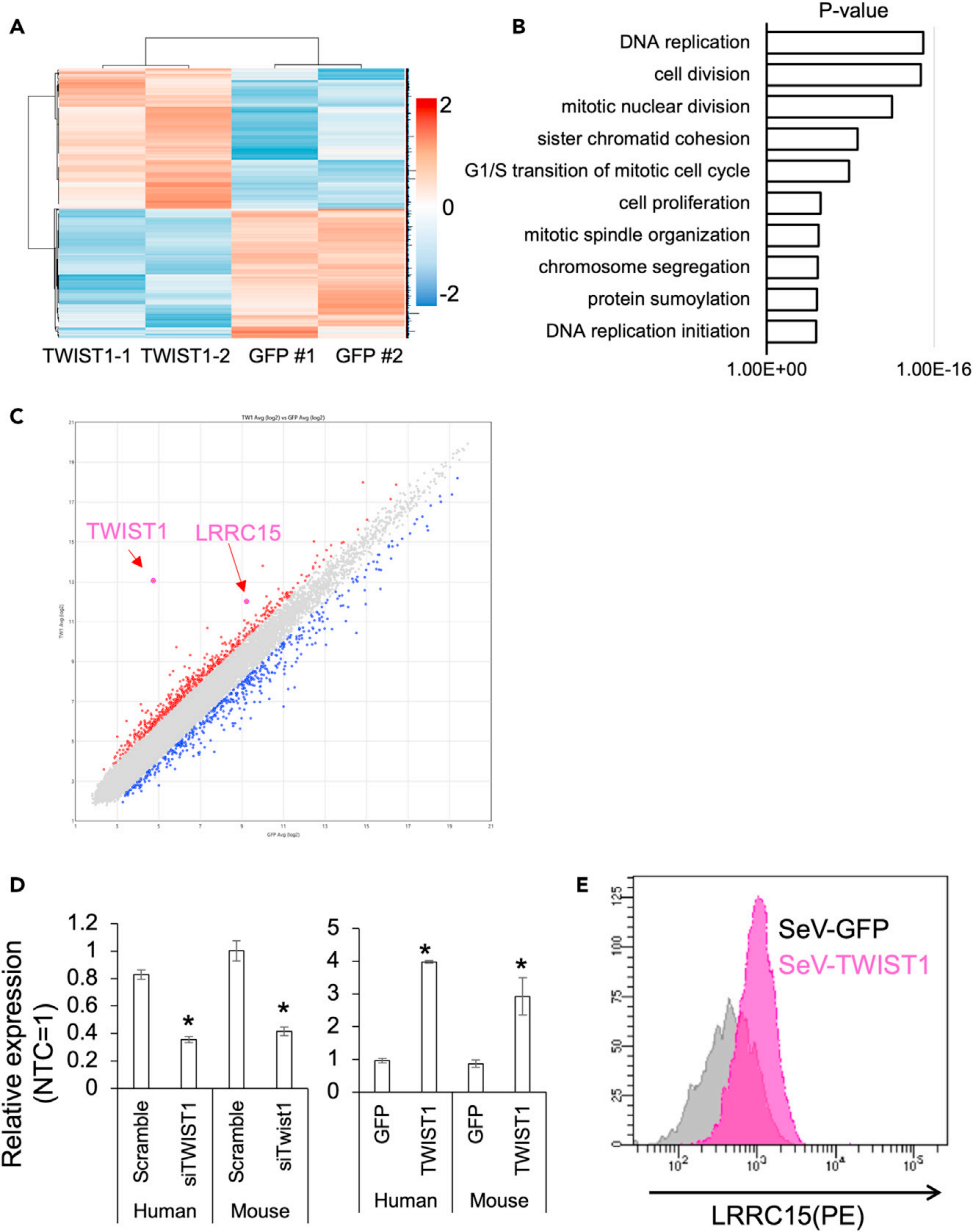
LRRC15 correlated with the expression of TWIST1 in human and mouse MSCs (A) Heatmap analysis with two independent samples as replicate. Common trends were observed between same experimental group. (B) Gene ontology (GO) enrichment analysis of differentially upregulated genes in the TWIST1 overexpressing human bone marrow MSCs. (C) Scatterplot analysis of differentially expressed genes in the human bone marrow MSCs overexpressing TWIST1 or GFP. (D) Expression changes of LRRC15 gene under different gene expression levels of TWIST1 in human and mice MSCs. Y axis show relative expression level to non-treatment control cells. Asterisk means significant difference detected at p < 0.05 (N = 3, biological replicates). (E) Increased expression of LRRC15 protein in the TWIST1-overexpressing human MSCs.

To evaluate the TWIST1-regulated stemness level in living cell, we searched for cell surface proteins that correlated with the TWIST1 expression. We analyzed the whole gene expression of two independent human MSC lines treated with SeV-TW1. Compared to the control MSCs treated with SeV-GFP, several genes showed different expression levels in SeV-TW1-transfected cells (Figure 4A). Gene Ontology (GO) enrichment analysis showed that nine of the top-10 GO terms were related to cell proliferation, indicating that the expression of TWIST1 is closely related to MSC proliferation and/or self-renewal (Figure 4B).

By analyzing the list of differentially expressing genes focusing on cell surface proteins that correlated with TWIST1 expression, we found that LRRC15 expression was significantly higher in the TWIST1-overexpressing MSCs (Figure 4C). To confirm that LRRC15 is associated with the expression level of TWIST1, we observed its expression in the MSCs treated with siTWIST1 or SeV-TW1 by qRT-PCR. Under the siTWIST1 treatment, gene expression of *Lrrc15/LRRC15* was downregulated to 40% of the control, and in contrast, the expression of *Lrrc15/LRRC15* was upregulated 3–4 times by SeV-TW1 introduction in both mouse and human MSCs (Figure 4D). Fluorescence-activated cell sorting (FACS) analysis showed that the cell surface expression of LRRC15 was upregulated by the overexpression of TWIST1 (Figure 4E).

### LRRC15-expressing population in mice bone marrow showed MSC characteristics

**Figure 5:**
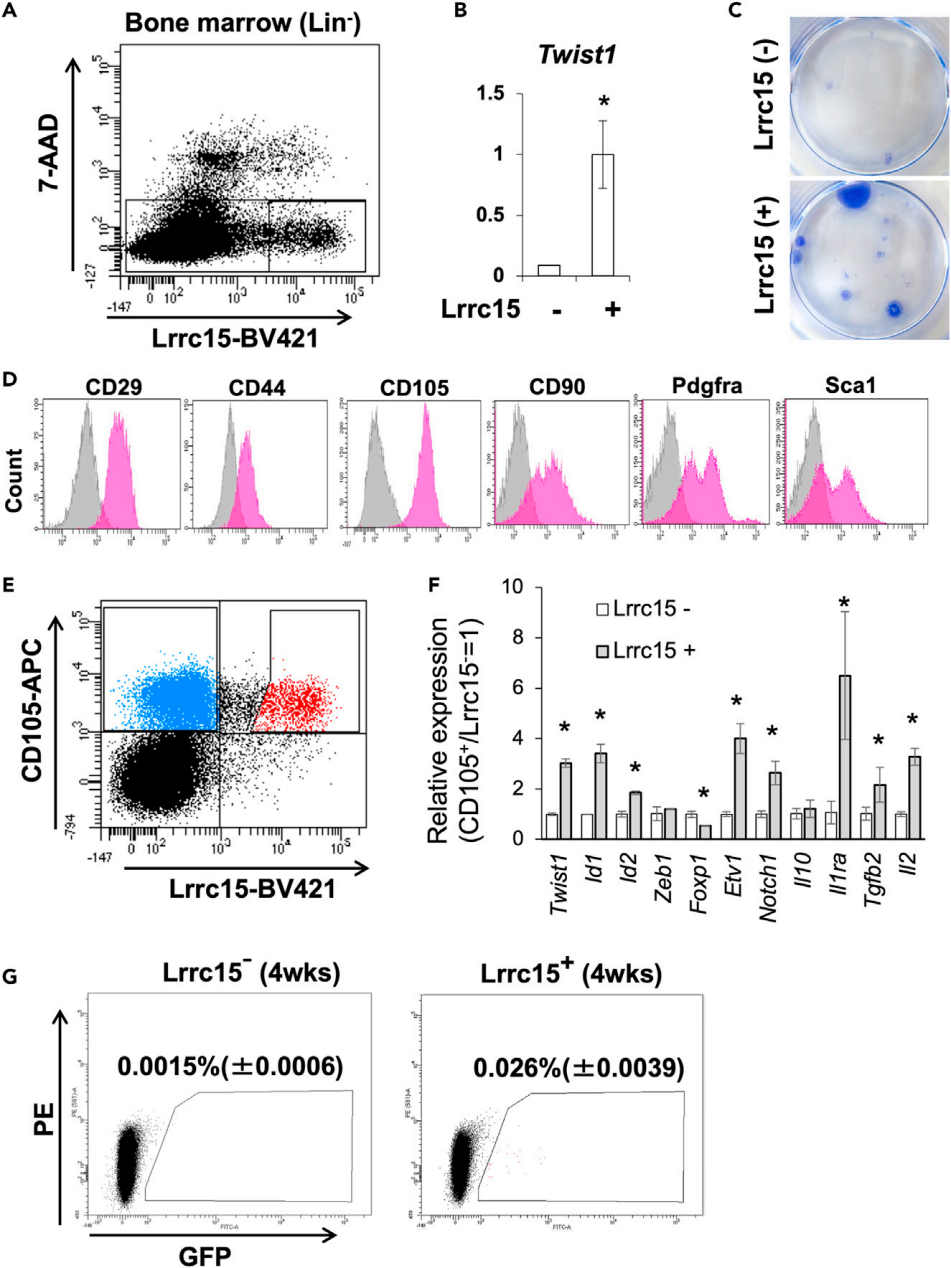
Characteristics of the LRRC15-positive cells in the mice bone marrow tissues (A) FACS analysis for the Lrrc15-expressing population in the CD11b/^−^CD45^-^/Ter119^-^/7-AAD^-^ fractions. (B) Comparison of *Twist1* gene expression level between the Lrrc15^-^ and Lrrc15^+^ populations. Asterisk means significant difference were detected at p < 0.05 (N = 6 from different animals). (C) Colony formation properties of the FACS-sorted cells from CD11b/^−^CD45^-^/Ter119^-^/7-AAD^-^/Lrrc15^-^ (upper) or CD11b^−^/CD45^-^/Ter119^-^/7-AAD^-^/Lrrc15^+^ (lower) fractions. (D) Flow cytometry analysis for the CD11b^−^/CD45^-^/Ter119^-^/7-AAD^-^/Lrrc15^+^ cells with the MSC markers in mice. Gray histograms show control cells treated with isotype antibodies, and pink histograms show samples treated with each antibody. (E) FACS sorting of the MSCs using anti-CD105 and anti-LRRC15 antibodies. (F) Comparing gene expressions of Twist1, stemness-associated transcription factors, and therapeutic cytokines in the CD11b^−^/CD45^-^/Ter119^-^/CD105^+^/Lrrc15^-^ and CD11b^−^/CD45^-^/Ter119^-^/CD105^+^/Lrrc15^+^ cells. Asterisks mean significant differences detected at p < 0.05 (N = 3, independent experiment). (G) Engrafted EGFP^+^ cells were detected by FACS analysis. Numbers in the figures are mean values with S.D. from three independent animals per each group.

Next, we examined whether Lrrc15-positive (LRRC15^+^) cells were present in the bone marrow and exhibited higher level of stemness than LRRC15^-^ cells. In bone marrow tissue of 6-week-old B6 mice, Lrrc15^+^ cells were present approximately in 5% of the viable non-hematopoietic cell populations that was separated as 7-AAD^-^/CD11b^−^/CD45^-^/Ter119^-^ (Figure 5A). The Lrrc15^+^ cells expressed Twist1 abundantly than the Lrrc15^-^ populations (Figure 5B) and showed higher colony-forming ability in *ex vivo* culture (Figure 5C). We observed expression of classic MSC markers in the Lrrc15^+^ populations and found that the Lrrc15^+^ cells expressed CD29, CD44, CD105, Pdgfra, and Sca1. For Pdgfra and Sca1, there were two populations with different expression levels (Figure 5D). The Lrrc15^-^ or Lrrc15^+^ MSCs identified as CD105^+^/Lrrc15^-^ and CD105^+^/Lrrc15^+^ were approximately 25% and 4% in the 7-AAD^-^/CD11b^−^/CD45^-^/Ter119^-^ non-hematopoietic population, respectively (Figure 5E). To characterize the Lrrc15^+^ MSCs in detail, we corrected CD105^+^/Lrrc15^-^ and CD105^+^/Lrrc15^+^ MSCs, and observed gene expression of the stemness-associated transcription factors and therapeutic cytokines.

In the CD105^+^/Lrrc15^+^ cells, both stemness-associated transcription factors *Twist1*, *Id1*, *Id2*, *Etv1*, and *Notch1* and therapeutic cytokines *IL1ra*, *Tgfb2*, and *Il2* were highly expressed compared to CD105^+^/Lrrc15^-^ cells. On the other hand, *Foxp1* expression was suppressed in Lrrc15^+^ MSCs (Figure 5F). To observe the homing properties of the Lrrc15^+^ MSCs, we transplanted CD105^+^/Lrrc15^-^ and CD105^+^/Lrrc15^+^ MSCs collected from B6-GFP donor mice into wild-type B6 recipients through tail vein. When we observed GFP-expressing donor cells by FACS at 4 weeks after cell transplantation, 0.0015% ± 0.0006% of Lrrc15^-^ MSCs and 0.026% ± 0.0039% of Lrrc15^+^ MSCs engrafted in the bone marrow (Figure 5G).

**Figure 6:**
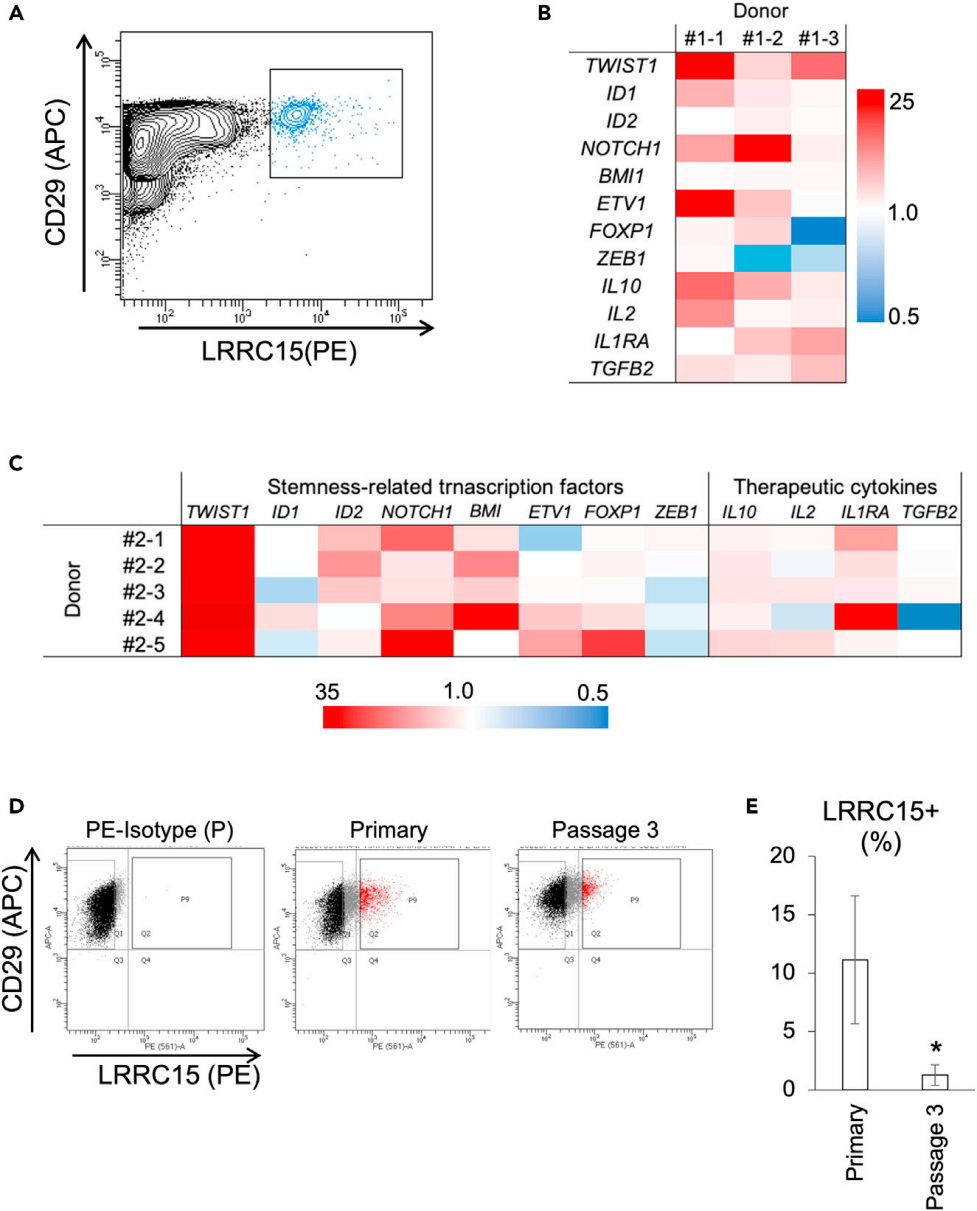
Characteristics of the LRRC15-positive cells in the human bone marrow tissues (A) FACS analysis showing the LRRC15^+^ population in the CD31^-^/CD45^-^/Ter119^-^/7-AAD^-^ and human MSC marker CD29^+^ fractions. (B) Comparing gene expressions of *TWIST1*, other stemness-associated transcription factors, and therapeutic cytokines between the CD31^-^/CD45^-^/Ter119^-^/CD29^+^/LRRC15^-^ and the CD31^-^/CD45^-^/Ter119^-^/CD29^+^/LRRC15^+^ MSCs. (C) Relative gene expression levels of the signature genes in the CD31^-^/CD45^-^/Ter119^-^/CD29^+^/LRRC15^+^ cells collected from primary cultures of 5 different donors. Heatmap shows relative expression to the CD31^-^/CD45^-^/Ter119^-^/CD29^+^/LRRC15^-^ cells in the primary culture of same donors each. (D) Decrease of the LRRC15^+^ population by *in vitro* culture. (E) Ratios of the LRRC15^+^ MSCs at primary and passage 3. Asterisk means significant difference detected at p < 0.05 (N = 5 from different donors).

We then examined whether LRRC15^+^ MSCs were present in the human bone marrow tissues. Since CD105^-^ subpopulations have been reported in humans,^14^ we used CD29 as an MSC marker and isolated the LRRC15^+^ MSCs as CD29 and LRRC15 double-positive populations (Figures 6A and S3). In human bone marrow tissues, CD29^+^/LRRC15^+^ MSCs existed 1.4 ± 1.0% in 7-AAD^-^/CD31^-^/CD45^-^/Ter119^-^ non-hematopoietic populations. Importantly, human LRRC15^+^ MSCs also showed higher expression of TWIST1 and stemness-associated transcription factors, except *FOXP1* and *ZEB1* these showed uneven pattern by donor (Figure 6B).

Next, we isolated the CD29^+^/LRRC15^+^ MSCs after primary culture to perform more reliable analysis minimizing the influence of a variety of times after harvest, patient background, and surgical procedure. In the five independent primary cultures from different donors, LRRC15^+^ MSCs expressed MSC markers CD73, CD90, and CD105 (Figure S4), and significantly higher *TWIST1* expression as well as stemness-related transcription factors, and therapeutic cytokines *IL1RA* and *IL10* than LRRC15^-^ MSCs (Figure 6C). The number of LRRC15^+^ MSCs decreased with passage: at passage 3, the rate of the CD29^+^/LRRC15^+^ population was reduced from approximately 10% to –2% (Figures 6D and 6E).

### Transplantation of the Lrrc15+ MSCs showed higher therapeutic effect in the pulmonary fibrosis model

**Figure 7:**
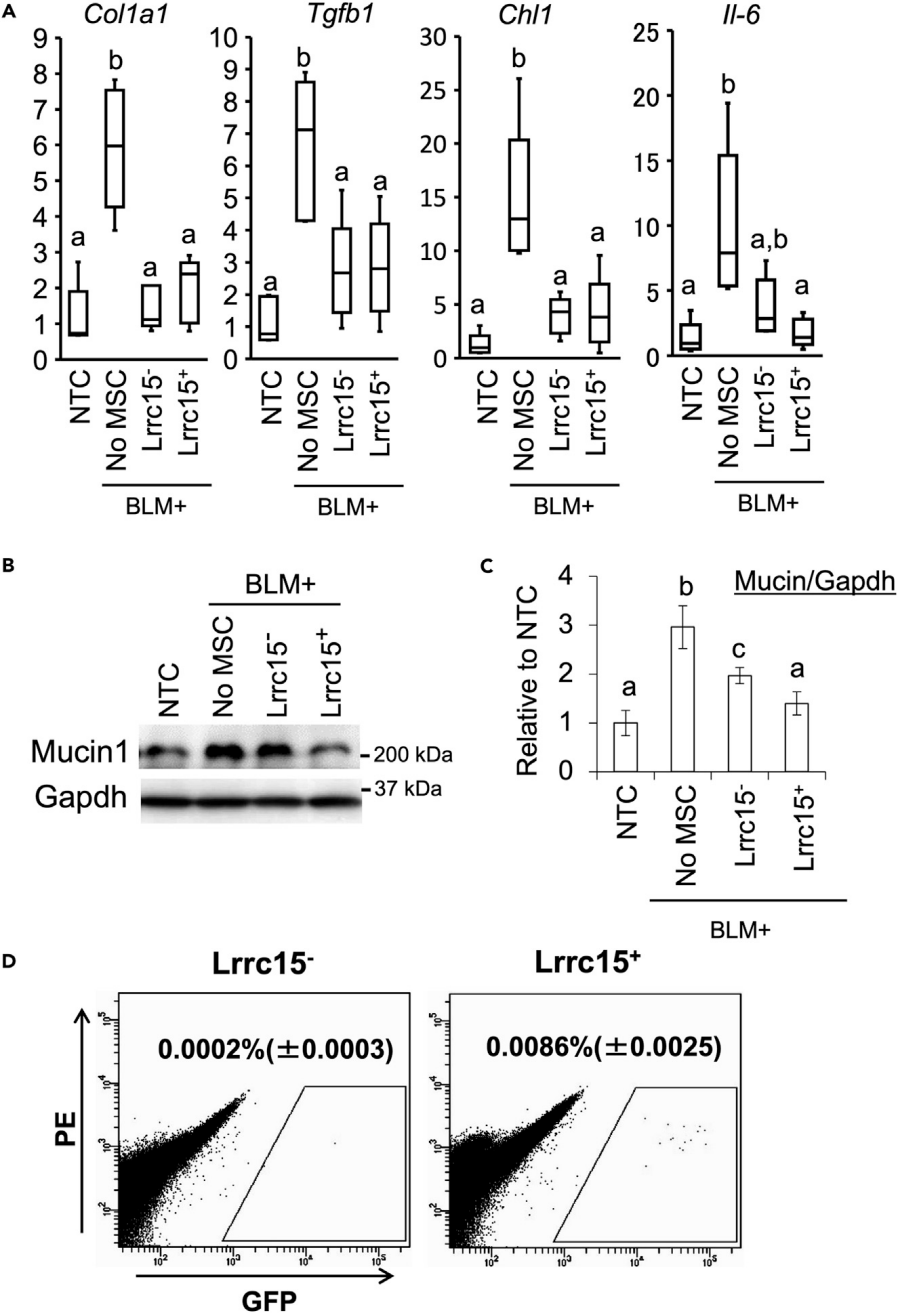
Therapeutic potentials of the Lrrc15-expressing cells in the bleomycin-induced pulmonary fibrosis (BPF) model mice (A) Expression changes of the inflammation/fibrosis markers in the BPF model mice on day 28 after cell transplantation. Asterisks mean significant difference detected at p < 0.05 (N = 5 from different animals). (B) Western blot for Mucin1 as inflammation/fibrosis markers. (C) Semi-quantitative analysis based on the western blot analysis of Mucin1. Blot of Mucin1 were divided by that of Gapdh and compared in each experimental group. Scores mean relative expression levels to non-treatment control (NTC). (D) Long-term engraftment of the transplanted cells in the injured lung of the model mice on day 28 (4 weeks) after transplantation. FACS analysis detected transplanted CD11b^−^/CD45^-^/Ter119^-^/CD29^+^/Lrrc15^-^ or Lrrc15^+^ MSCs of B6-EGFP donor mice. Numbers in the figures are mean values with S.D. from three independent animals per each group.

To investigate whether Lrrc15^+^ MSCs exhibit therapeutic potential for cell transplantation, we prepared a pulmonary fibrosis (PF) model by intratracheal injection of bleomycin. After 24 h of bleomycin injection, 1 × 10^5^ CD105^+^/Lrrc15^+^ MSCs were isolated from donor B6-GFP mouse bone marrow and transplanted into the tail vein of the PF model mice. After 4 weeks of the bleomycin injection, qRT-PCR was performed on the lung tissue of the model mice. Bleomycin injection significantly increased the expression of fibrosis markers *Col1a1*, *Tgfb1*, *Chl1*, and *Il6*. In both groups in which CD105^+^/Lrrc15^-^ and CD105^+^/Lrrc15^+^ MSCs were transplanted, the expression of *Col1al1*, *Tgfb1*, and *Chl1* was significantly decreased. Histological analysis with Masson’s Trichrome staining showed that accumulation of collagen fibers occurred in the lung of bleomycin-treated model mice, and the fibrotic changes were suppressed in the MSC-transplanted mice (Figure S5). As for the gene expression of *Il6*, statistically significant suppression was observed in model mice transplanted with CD105^+^/Lrrc15^+^ MSCs (Figure 7A). To determine whether Lrrc15^+^ MSCs were more effective transplants for the treatment of PF than Lrrc15^-^ MSCs, we quantified Mucin1, which is an indicator of inflammation and fibrosis in the lungs. Mucin1 expression was markedly upregulated following bleomycin administration. Although both Lrrc15^-^ and Lrrc15^+^ MSCs transplantation resulted in decreased Mucin1 expression, the suppressive effect of Mucin1 was more prominent in the lungs of mice transplanted with Lrrc15^+^ MSCs than in those transplanted with Lrrc15^-^ MSCs (Figures 7B and 7C). FACS analysis of the lungs of model mice revealed that 0.0002% ± 0.0003% of transplanted Lrrc15^-^ MSCs and 0.0086% ± 0.0025% of LRRC15^+^ MSCs settled in the lungs of model mice 4 weeks after cell transplantation (Figure 7D).

## Discussion

MSCs are currently being explored as useful cell source for cell transplantation therapies for various diseases.^15^ However, key transcription factors essential for maintenance of stemness and reliable markers to evaluate its stemness are largely unknown in MSCs. In this study, we focused on TWIST1, which regulates mesenchymal tissue development,^16^^,^^17^ as a key transcription factor that maintains the stemness of MSCs. Several studies have suggested TWIST1 involvement in epithelial-mesenchymal transition (EMT)-mediated metastasis and malignant transformation of cancer, and TWIST1 expression levels have been found to correlate with patient prognosis.^18^^,^^19^^,^^20^^,^^21^^,^^22^ As a classical transcription factor, TWIST1 is involved in the regulation of cadherin-mediated cell attachment, cell proliferation, and the acquisition of drug resistance.^23^^,^^24^^,^^25^ Recently, several groups, including ours, have reported that TWIST1 is also involved in epigenetic modification.^26^^,^^27^ The effect of TWIST1 on cell fate determination is strong, as shown by Mani et al., who found that only TWIST1 introduction can change the cell lineage of mammary epithelial cells to MSC-like cells and provide multiple differentiation potentials.^28^^,^^29^ This evidence suggests that TWIST1 is deeply involved in the stemness as a key transcription factor. Furthermore, Boregowda et al. developed a quality rating scale for MSCs based on TWIST1 expression levels, and showed that it is possible to predict the clinical effectiveness of MSC by the TWIST1 expression level.^13^ This suggests that it is highly rational to evaluate MSC quality using expression level of TWIST1.

In this study, we first observed changes in *TWIST1* expression level in the senescent MSCs induced by repetitive passages *in vitro*. In our experiments, after 10 passages in humans and 6 in mice, MSCs showed senescence-specific morphology and lost proliferation properties. In the senescent MSCs, expression level of *TWIST1* significantly decreased,^12^ and expression levels of other stemness-associated transcription factors *ID1*, *ID2*, *BMI1*, *ZEB1*, *NOTCH1*, *FOXP1*, and *ETV1* were also suppressed. We used these transcription factors to evaluate the stemness of MSCs since they are reported to be essential for stem cell characteristics of somatic stem cells including MSCs: ID1 and ID2 are family members of inhibitors of DNA binding (IDs), which are abundantly expressed in proliferating tissues, including embryonic and adult stem cell populations, and can maintain the stemness of various somatic stem cells.^30^^,^^31^ Bmi1, a polycomb group repressor, is an essential transcription factor for the self-renewal of somatic stem cells, including hematopoietic stem cells and neuronal stem cells. In MSCs, Bmi1 regulates proliferation, multipotency, and senescence.^32^^,^^33^ Zeb1 and Notch1 are transcription factors essential for stemness and have been demonstrated to regulate the self-renewal and undifferentiated status of stem cells.^34^^,^^35^ FOXP1 attenuates MSC senescence by orchestrating the cell fate switch while maintaining their replicative capacity.^36^ It has also been demonstrated that overexpression of FOXP1 increased promoter activity of other stemness-associated molecules such as ABCG2, OCT4, NANOG, and SOX2.^37^ The ETS family transcription factor ETV1 is an important molecule for the proliferation of gastrointestinal stromal tumor cells.^38^ Kubo et al. reported that both FoxP1 and Etv1 are involved in the stemness of MSCs and are useful markers for MSC characterization.^39^

We also determined that suppression of *TWIST1* by siRNA resulted in reduced proliferation and migration, as previous evidence has shown that TWIST1*-*silenced bone marrow MSCs were slower to reach confluence during expansion culture.^40^ Decreased expression of *TWIST1* clearly affected the expression of stemness-associated transcription cytokines important for the therapeutic properties of MSCs. In contrast, *TWIST1* overexpression increased cell proliferation and the expression of *ID1*, *ID2*, *BMI1*, and *ZEB1*, in addition to the EMT-related genes (Figure S6). Furthermore, the expression of the therapeutic cytokines *IL1Ra* and *IL10* was also upregulated by *TWIST1* overexpression. These results were partly consistent with the previous reports that Twist2, which is another subfamily of Twist1 and has high homology and overlapping expression pattern with Twist1, promotes the expression of IL10 directly.^41^ While the TWIST1 can promote expression of therapeutic cytokines, it has been demonstrated that Twist1 and Twist2 modulate the expression of proinflammatory cytokines through inhibition of NF-κB-mediated transactivation.^41^^,^^42^ These notions indicate that forced expression of *TWIST1* increased the stemness and therapeutic effect of MSCs, and application of *TWSIT1*-overexpressing MSCs for cell therapy was thought to be highly rational. However, there are at least two challenges in transplanting *TWSIT1*-overexpressing MSCs for therapeutic purposes. First, *TWIST1*-overexpressing cells are impaired in differentiation both *in vivo* and *in vitro*. It has been reported that TWIST1 inhibits differentiation to osteoblast^12^^,^^43^ and chondrocyte.^44^ Also in the present study, *TWIST1*-overexpressing MSCs showed lower efficiency in *in vitro* differentiation compared to MSCs transfected with a GFP-cording SeV (Figure S7). Second, numerous studies have shown that TWIST1 can function as an oncogenic factor. TWIST1 expression induces malignant properties such as initiation, stemness, and invasion of cancer.^45^ Thus, it is necessary to develop a system to completely control exogenous TWIST1 expression if we plan to apply TWIST1-overexpressing MSCs to cell therapy.

On the other hand, the fact that TWIST1 expression tightly correlates with stemness of MSCs suggests that TWIST1 can be useful at least as an indicator to measure the stemness and/or quality of MSCs, as reported previously.^13^ Since TWIST1 protein is located only in the cytoplasm and nucleus, its expression/activity cannot be quantified without destroying the cells. To clarify this dilemma, we examined cell surface molecules that correlate with TWSIT1 expression and found two cell surface molecules, TNFRSF10D and LRRC15 by microarray analysis for the *TWIST1*-overexpressing MSCs. Of the two candidate proteins, we decided to examine the LRRC15 protein as a marker. LRRC15 is a member of the LRR superfamily and has been reported to be involved in cell-cell interactions and metastasis.^46^ LRRC15 is not normally expressed in most normal tissues but is found in hair follicles, tonsils, stomach, spleen, osteoblasts, and wound healing sites.^47^ Importantly, MSCs have been found in all tissues described previously,^48^^,^^49^^,^^50^^,^^51^ and Purcell et al. demonstrated that LRRC15 is also expressed in cultured bone marrow MSCs.^47^ These notion means that LRRC15 could be used as a specific marker for stemness possessing cells. In addition, gene expression correlation with that of TWIST1 is also observed in the bladder cancer thyroid carcinoma and pancreatic cancers, when analyzed using TCGA dataset (Figures S8 and S9). Based on the results of our microarray analysis and the above evidence, we hypothesized that LRRC15 is a promising marker for identifying MSCs expressing TWIST1 at high level.

Consistent with our hypothesis, LRRC15 expression was linked to TWIST1 expression in both mice and human MSCs; *Lrrc15/LRRC15* expression was decreased by treatment with *Twsit1/TWIST1* siRNA. In contrast, TWIST1 overexpression increased *Lrrc15/LRRC15* expression. Native elongating transcript–cap analysis of gene expression analysis^52^ using TWIST1-overexpressing human dermal fibroblast cells also showed that TWIST1 overexpression activates transcription of nascent LRRC15 RNA (Figure S10). These results support that LRRC15 is directly regulated by TWIST1, and its expression may be useful to speculate TWIST1 activity in the MSCs.

In mouse bone marrow tissues, Lrrc15^+^ populations accounted for approximately 5% of all non-hematopoietic cells, and expressed classical MSC markers CD29, CD105, and CD90, means that the Lrrc15^+^ population was a part of MSCs.

Interestingly, the Lrrc15^+^ MSC population included cells showing different Pdgfra and Sca1 expression levels. Pdgfra^+^/Sca1^+^ (PαS^+^) are markers that can identify undifferentiated MSCs.^8^ In our observations, Lrrc15^+^ cells were present in approximately 50% of the PαS^+^ population, indicating that the PαS^+^ population and Lrrc15^+^ were not identical. PαS^+^ cells are characterized by the expression of PDGF receptor alpha and glycosyl phosphatidylinositol-anchored cell surface protein Sca1. On the other hand, the Lrrc15^+^ represents Twist1-expressing MSCs that is thought to be dynamic cellular state accompanied migration and proliferation compared to PαS cells. In other words, the target populations may be slightly different between PαS^+^ and Lrrc15^+^, and the overlap ratio between PαS^+^ and Lrrc15^+^ may vary depending on MSC activity or probably donor conditions. More detailed analysis, including a single-cell-level analysis, will be necessary to determine which of the two methods may identify stem cells at a higher level of hierarchy.

We observed that Lrrc15^+^ MSCs highly expressed the stemness-associated genes and could repopulate into bone marrow tissues. These results clearly show that Lrrc15^+^ MSCs possess high levels of stemness than the Lrrc15^-^ population. Importantly, LRRC15^+^ cells were also found in human bone marrow tissues, and showed higher expression of stemness-associated transcription factors, including *TWIST1* and therapeutic cytokines. Unlike in mice, the percentage of LRRC15^+^ MSCs was very low, and there was large quantitative divergence between donors. In human specimens, a variety of factors could affect gene expression status, not only donor disease, inflammation, and age but also the surgical procedure and hemorrhagic condition during surgery. To observe differences in stem cell characteristics between LRRC15^-^ and LRRC15^+^ cells reducing these factors, we performed primary culture followed by FACS. LRRC15^+^ cells were present in approximately 5%–15% of CD29^+^ adherent cells in the primary culture and showed higher expression of *TWIST1* and stemness-associated transcription factors and therapeutic cytokines than the LRRC15^-^ population. Under both *in vivo* and *in vitro* conditions, not all donors showed higher expression of all signature genes in LRRC15^+^ MSCs, although *TWIST1* was more than 20-fold higher in LRRC15^+^ cells in all cases. This inconsistency is likely because the present observation was based on a relative comparison between LRRC15^+^ and LRRC15^-^ MSCs, in which if LRRC15^-^ main populations retain sufficient MSC properties, the difference between LRRC15^+^ and LRRC15^-^ may be relatively small or reversed if the main population includes subpopulations that strongly express certain genes.

*In vitro* analysis using primary MSCs revealed that the rate of LRRC15^+^ MSCs clearly decreased with passaging, indicating that the quality of MSCs is degraded by passaging, as shown in numerous previous studies, and that it is difficult to expand an MSC population with high levels of LRRC15/TWIST1 under normal culture conditions. At the same time, this result suggests that it is possible to develop appropriate culture conditions by focusing on LRRC15 expression.

To further examine whether LRRC15^+^ MSCs possess therapeutic function, we performed cell transplantation in a bleomycin-induced pulmonary fibrosis (BPF) model, which is one of the most established drug-inducible disease models. There are numbers of evidence for MSC-based therapy in the pulmonary fibrosis.^53^^,^^54^ Consistent with previous studies, MSC transplantation suppressed the expression of fibrosis marker genes in the lungs of the BPF mice. Furthermore, as expected, transplantation of Lrrc15^+^ MSCs showed a stronger suppressive effect on *Il6* and Mucin1 expression than Lrrc15^-^ MSCs. Importantly, Lrrc15^+^ MSCs remained in the injured lungs 4 weeks after transplantation, unlike the case of Lrrc15^-^ cell transplantation.

This difference in engraftment efficiency between Lrrc15^-^ and Lrrc15^+^ cells was thought to be due to differences in tolerance to apoptosis and migration properties. TWIST1 is involved in epithelial-mesenchymal transition, cell migration, and metastasis. These properties are important for homing and repopulation of transplanted cells in the injured tissues. In addition, TWIST1 suppresses MYC- and p53-dependent apoptosis.^21^ These characteristics are advantageous for transplanted cells, and the selection of LRRC15-positive cells for transplantation is likely to result in more effective therapy.

However, it is still unclear which hierarchy of MSCs is labeled by LRRC15, and whether LRRC15^+^ MSCs are the most competent cell source for various diseases. There is evidence that LRRC15 is upregulated during osteoblast differentiation of MSCs and functions as a repressor of NF-κB by promoting the nuclear exclusion of p65.^55^ These observations appear to be inconsistent with our hypothesis that LRRC15 is a marker of undifferentiated MSC populations. As an explanation for this discrepancy, Wang et al. observed expression changes in LRRC15 using expanded MSCs *in vitro*, in which LRRC15 expression should be dramatically suppressed compared to primary MSCs. Furthermore, it has been demonstrated that LRRC15 expression is upregulated by Tgf*β* signaling.^47^ Wang et al. used dexamethasone, which is known to activate the Tgf*β* pathway,^56^ for osteoblast differentiation. Thus, it is also possible that LRRC15 levels were elevated by dexamethasone administration *in vitro*. It is thought that comparing the expression change of LRRC15 between MSCs during osteoblast differentiation and FACS-sorted MSCs from bone marrow tissues or primary MSCs will provide a clear answer to this discrepancy.

In addition, we may need to pay attention to the intrinsic function of LRRC15 expression itself in using LRRC15 as a novel marker for MSCs. Although the detailed function of LRRC15 and its role in stem cells is largely unknown, some findings suggest its role relating the stem cell function such as proliferation and invasion.^57^^,^^58^ Mechanistically, it has been found that LRRC15 promotes metastasis upon interaction with fibronectin and β1 integrin, leading to activation of focal adhesion kinase signaling.^47^^,^^59^ Furthermore, Yang et al. demonstrated that suppression of LRRC15 by siRNA treatment induced reduction of MYC and cyclin D1 expression in human breast cancer cell lines.^60^ Consistent with this observation, we observed that siRNA-mediated suppression of LRRC15 in the hMSCs led to decreased expression of MSC and cell proliferation-related genes PCNA and CCNB1 (Figure S11). Interestingly, TWIST1 expression was also affected by the LRRC15 suppression. Although the present study did not examine about detailed mechanisms how LRRC15 involved in regulation of TWIST1 expression, Selmi et al. have demonstrated that TWSIT1 is directly regulated by MYC.^61^ This means that LRRC15 is not merely a marker to predict TWIST1 expression, but is itself may involve in regulation of stemness.

For the complete validation of whether TWIST1- and LRRC15-expression is useful as a marker for MSCs, it will be essential to clarify the location of TWIST1/LRRC15 expressing cells *in vivo* and to determine their physiological functions. This knowledge may also lead to improved methods for MSC culture.

As a conclusion, we demonstrated three notions: 1) TWIST1 is a key factor for stemness of MSCs both in humans and mice; 2) its activity can be evaluated by expression of the cell surface protein LRRC15; and 3) LRRC15^+^ MSCs show higher expression of some stemness-associated transcription factors, including TWIST1, and exhibit superior therapeutic potentials than LRRC15^-^ MSCs.

Our results indicate that LRRC15 may be an optimal marker for evaluating MSC stemness and therapeutic efficacy. The clear advantage of LRRC15 expression-based evaluation of MSCs is their common availability in mice and humans. This advantage allows for easy extrapolation of the results from experiments in model animals and could reveal many unknown properties and characteristics of MSCs.

### Limitations of the study

The reversibility of LRRC15 expression remains unclear: present study demonstrated that LRRC15 is a valid marker for undifferentiated MSCs with dynamic properties supported by TWIST1. On the other hand, we do not have enough information to conclude the LRRC15-negative MSCs as cell populations with reduced stemness.

It is necessary to determine whether the LRRC15/TWIST1-positive MSCs can emerge from the LRRC15-negative populations, and if so, it is also essential to identify what cell populations among them have such plasticity. Furthermore, gene expression of LRRC15 and TWIST1 clearly does not correlate in several cancer types such as adrenocortical carcinoma or melanoma. Whether the relation between LRRC15 and TWIST1 is conserved in other stem cells or specific characteristics in mesenchymal cell types will also have to be clarified in future studies.

The differentiation directivity of LRRC15 stem cells also should be examined: If the LRRC15-positive MSCs may have some biased differentiation capacity, this could be a characteristic to consider as a limitation or risk for clinical application. The accumulation of such information is essential for the realization of safe and high-quality cell transplantation.

## STAR★Methods

### Key resources table

**Table:** 

REAGENT or RESOURCE	SOURCE	IDENTIFIER
**Antibodies**		

Rabbit polyclonal anti-LRRC15 antibody	Bioss	Cat# bs-6815R; RRID: AB_11117937
Samortamab anti -LRRC15	Proteogenix	PX-TA1518-100UG
Bv421 Donkey anti-rabbit IgG	Biolegend	Cat# 406410 (Poly4064); RRID: AB_10897810
CD11b-FITC	TONBO	Cat# 35-0112 (M1/70); RRID: AB_2621676
TER119-FITC	TONBO	Cat# 35-5921 (TER-119); RRID: AB_2621720
CD45-FITC	Biolegend	Cat# 103108 (30-F11); RRID: AB_312973
CD140a-APC	Biolegend	Cat# 135908 (APA5); RRID: AB_2043970
CD29-APC	Biolegend	Cat# 102216 (HMβ1-1); RRID: AB_492833
CD105-APC	eBioscience	Cat# 17-1051-82 (MJ7/18); RRID: AB_2573155
CD44-PE	TONBO	Cat# 50-0441 (IM7); RRID: AB_2621762
CD90.2-PE	eBioscience	Cat# 12-0902-82 (53-2.1); RRID: AB_465776
Sca1-PE eFluor 610	invitrogen	Cat# 61-5981-82 (D7); RRID: AB_2574648
CD45-FITC	Biolegend	Cat# 368508 (2D1); RRID: AB_2566368
CD31-FITC	Biolegend	Cat# 303104(WM59); RRID: AB_314330
CD29-APC	Biolegend	Cat# 303008 (TS2/16); RRID: AB_314324
Mouse monoclonal anti - Mucin1 antibody	Santacruz	Cat# sc-53381(SM3); RRID: AB_628990
Mouse monoclonal anti - PCNA antibody	Cell Signaling	Cat# 2586(PC10); RRID: AB_2160343
Rabbit Polyclonal anti - TWIST1 antibody	Cell Signaling	Cat# 46702; RRID: AB_2799308
Rabbit Polyclonal anti - P16INK4A antibody	SIGMA	Cat# SAB4500072; RRID: AB_10743680
Mouse monoclonal anti - Gapdh antibody	FUJIFILM	Cat# 015-25473, RRID: AB_2665526

**Oligonucleotides**		

*Mouse Twist1*	Eurofin genomics	5′-AGTCGTACGAGGAGCTGCAG 5′-AACGCCTCGTTCAGCGACTG
*Mouse Id1*	Eurofin genomics	5′-AGCTGAACTCGGAGTCTGAAG 5′-TCAGCGACACAAGATGCGATC
*Mouse Id2*	Eurofin genomics	5′-TGAACACGGACATCAGCATCC 5′-AGCCACAGAGTACTTTGCTATC
*Mouse Bmi1*	Eurofin genomics	5′-TTTATGCAGCTCACCCGTC 5′-CTCCTCATCTGCAACTTCTCC
*Mouse Zeb1*	Eurofin genomics	5′-AGAGCACTTACGGATTCACAG 5′-TGAGCTATAGGAGCCAGAATG
*Mouse Notch1*	Eurofin genomics	5′-TGGACAAGATCAATGAGTTC 5′-ACACTCATCCACATCATACTG
*Mouse FoxP1*	Eurofin genomics	5′-AGGAATGACAAGCAACCAGCTC 5′-TGTTGAGGAGTGATAACCTGAG
*Mouse Etv1*	Eurofin genomics	5′-AAGTGCCTGTACAATGTCAG 5′-ACTGGAGTGCTGGATGGTGTC
*Mouse IL2*	Eurofin genomics	5′-TACAGGAGCTCCTGAGCAG 5′-TTGAAGGTGAGCATCCTGG
*Mouse IL10*	Eurofin genomics	5′-TGCTAACCGACTCCTTAATGC 5′-ATCATTTCCGATAAGGCTTGG
*Mouse IL1Ra*	Eurofin genomics	5′-TCATTGCTGGGTACTTACAAGG 5′-AGAACACACTATGAAGGTCAATAG
*Mouse Tgfb2*	Eurofin genomics	5′-AATCTGGTGAAGGCAGAGTTCAG 5′-ACAACCTTGCTATCGATGTAGCG
*Mouse Tgfb1*	Eurofin genomics	5′-AGCCTGGACACACAGTACAG 5′-TGTGTTGGTTGTAGAGGGCAAG
*Mouse Col1a1*	Eurofin genomics	5′-TGGACTTCCTGGTCCTCCTG 5′-ATCATAGCCATAGGACATCTGG
*Mouse Chl1*	Eurofin genomics	5′-TAGACTAGGAACTGCAGTATCAG 5′-TGCAGGGTAAGACAATGGAATC
*Mouse IL6*	Eurofin genomics	5′-AGGCTTAATTACACATGTTCTCTG 5′-TCATCGTTGTTCATACAATCAG
*Mouse Lrrc15*	Eurofin genomics	5′-AACACACACATCACCGAACTC 5′-AGATTGCGGAAGGCACCTGG
*Mouse Gapdh*	Eurofin genomics	5′-TCGTGGAGTCTACTGGTGTC 5′-TCGTGGTTCACACCCATCAC
*Human TWIST1*	Eurofin genomics	5′-TCGAGAGATGATGCAGGACG 5′-TCTTCCTCGCTGTTGCTCAG
*Human ID1*	Eurofin genomics	5′-ATCGACTACATCAGGGACC 5′-TCGGATCTGGATCTCACCTC
*Human ID2*	Eurofin genomics	5′-ACCACCCTCAACACGGATATC 5′-TCAGCCACACAGTGCTTTGCTG
*Human NOTCH1*	Eurofin genomics	5′-AGGAAACAACTGCAAGAACG 5′-TGGCACTCGTCCACATCCTC
*Human BMI1*	Eurofin genomics	5′-AGAAGGGATTTTTATGCAGC 5′-TTCATCTGCAACCTCTCCTC
*Human ETV1*	Eurofin genomics	5′-TGCCTGCAGTCAAGAACAGC 5′-ACTTGTGGCTTCTGATCATAG
*Human FOXP1*	Eurofin genomics	5′-TGGACAGCTCTCAGTCCACAC 5′-TGCATACACCATGTCCATAGAG
*Human ZEB1*	Eurofin genomics	5′-AGAGCACTTAAGAATTCACAG 5′-TGCAGTTTGGGCATTCATATG
*Human IL10*	Eurofin genomics	5′-ATCAAGGCGCATGTGAACTC 5′-TTCACATAGCCTTGTCCTAC
*Human IL2*	Eurofin genomics	5′-ACAACTGGAGCATTTACTGCTG 5′-TGTGAGCATCCTGGTGAGTTTG
*Human IL1RA*	Eurofin genomics	5′-TACTTGCAAGGACCAAATGTC 5′-AAGAACAGAGCATGAGGCTC
*Human TGFB2*	Eurofin genomics	5′-TGAACAACGGATTGAGCTATATC 5′-ATGTAGCGCTGGGTTGGAGATG
*Human LRRC15*	Eurofin genomics	5′-AGATCCTCAACACGCACATCAC 5′-CTCATTCTTCTCAATCCTCAGG
*Human GAPDH*	Eurofin genomics	5′-TGGTAAAGTGGATATTGTTGC 5′-TTCTCAGCCTTGACGGTGC

**Software and algorithms**		

*cBioPortal*	Memorial Sloan Kettering Cancer Center	https://www.cbioportal.org
*JMP 17*	JMP Statistical Discovery LLC.	

**Deposited data**		

*Bladder Cancer (MSK/TCGA, 2020)*	TCGA	https://www.cbioportal.org/study?id=blca_msk_tcga_2020
*Bladder Urothelial Carcinoma (TCGA, PanCancer Atlas)*	TCGA	https://www.cbioportal.org/study?id=blca_tcga_pan_can_atlas_2018
*Rectal Cancer (MSK, Nature Medicine 2022)*	TCGA	https://www.cbioportal.org/study?id=rectal_msk_2022
*Colorectal Adenocarcinoma (TCGA, PanCancer Atlas)*	TCGA	https://www.cbioportal.org/study?id=coadread_tcga_pan_can_atlas_2018
*Esophageal Adenocarcinoma (TCGA, PanCancer Atlas)*	TCGA	https://www.cbioportal.org/study?id=esca_tcga_pan_can_atlas_2018
*Lung Adenocarcinoma (TCGA, PanCancer Atlas)*	TCGA	https://www.cbioportal.org/study?id=luad_tcga_pan_can_atlas_2018
*Pancreatic Adenocarcinoma (TCGA, PanCancer Atlas)*	TCGA	https://www.cbioportal.org/study?id=paad_tcga_pan_can_atlas_2018
*Pancreatic Adenocarcinoma (TCGA, Firehose Legacy)*	TCGA	https://www.cbioportal.org/study?id=paad_tcga
*Mesothelioma (TCGA, PanCancer Atlas)*	TCGA	https://www.cbioportal.org/study?id=meso_tcga_pan_can_atlas_2018
*Sarcoma (TCGA, PanCancer Atlas)*	TCGA	https://www.cbioportal.org/study?id=sarc_tcga_pan_can_atlas_2018
*Thyroid Carcinoma (TCGA, PanCancer Atlas)*	TCGA	https://www.cbioportal.org/study?id=thca_tcga_pan_can_atlas_2018
*Kidney Renal Clear Cell Carcinoma (TCGA, PanCancer Atlas)*	TCGA	https://www.cbioportal.org/study?id=kirc_tcga_pan_can_atlas_2018

### Resource availability

#### Lead contact

Further information and requests for resources and reagents should be directed to and will be fulfilled by the lead contact, Takeshi Teramura (teramura@med.kindai.ac.jp).

#### Materials availability

This study did not generate new unique reagents and materials.

### Experimental model and study participant details

#### Ethics statement

The Institutional Review Board in Kindai University Faculty of Medicine approved the human tissue procurement protocol. Tissue samples were harvested from patients who underwent total hip arthroplasty, total knee arthroplasty, and anterior cruciate ligament reconstruction after obtaining informed consent at Kindai University Hospital.

All animal handling, care, operation, and sacrifice procedures were approved by the Institutional Animal Care and Use Committee of Kindai University and were performed in accordance with institutional guidelines and regulations.

C57BL/6J mice (B6, 6-week-old, male) were procured from CLEA Japan for conducting experiments. The mice were provided *ad libitum* access to food and water and were maintained on a 12-hour light in the standard rodent cage. To prepare the bleomycin-induced pulmonary fibrosis (BPF) model for cell transplantation experiments, B6 mice were anesthetized using 2% isoflurane. Following anesthesia, pulmonary fibrosis was induced by intratracheal instillation of BLM (2.0 mg/kg, Nippon Kayaku, Japan) in 20 μL phosphate-buffered saline (PBS).

### Method details

#### Isolation and culture of mouse BMMSCs

Six-week-old B6 male mice were used for the animal experiments. To establish mouse BMMSCs, the cleaned femurs and tibias were cut into small pieces and treated with 0.1% collagenase type II (Wako, Tokyo, Japan) for 15 min. Single-cell suspensions containing BMMSCs were collected, washed twice with PBS, and plated onto cell culture dishes (Sumilon, Sumitomo Bakelite Co. Ltd., Tokyo, Japan) in αMEM (Thermo Fisher Scientific, Waltham, MA, USA) supplemented with 200 mM L-glutamine and 10% fetal bovine serum (CORNING, Lowell, MA, USA) under 5%CO_2_ and 5%O_2_ at 37 °C. The medium was replaced the following day to remove dead cells and debris. After 10 days of culture, BMMSCs were disaggregated using Accutase (Nacalai tesque, Kyoto, Japan) and evaluated by FACS using PDGFRα, CD105, and Sca1 antibodies. Only the cells showing triple positivity for the markers over 95% of cells and passaged once or twice were used in subsequent experiments.

#### Isolation and culture of human MSCs

Bone marrow fluids were collected from patients who provided explanations using documents and agreed to donate materials prior to surgery. Using a 50 mL syringe and an 18G needle, 5 mL of bone marrow fluid was aspirated from the exposed bone marrow tissues during surgery. The collected bone marrow fluids were then centrifuged and treated with collagenase type I for 15 min. Digested tissues were filtered with a CellStrainer, centrifuged, washed twice with 10%FCS-αMEM, and plated onto cell culture dishes in 10%FCS-αMEM under 5%CO_2_ and 5%O_2_ at 37 °C. On day 2 of culture, the medium was replaced to remove dead cells and debris. After 10 days, BMMSCs that formed colonies of fibroblastic cells were disaggregated using TrypLE Express and evaluated by FACS using CD105 and CD44 antibodies. Only the cells showing double positivity for the markers over 95% of cells and passaged once or twice were used in subsequent experiments.

#### Production and infection of sendai virus (SeV) vectors

cDNA encoding human TWIST1 and GFP was prepared using PCR. The preparation of vector packaging cells and production of SeV vectors were performed according to the methods we previously established.^62^^,^^63^ Cells were infected with SeV vectors at a multiplicity of infection (MOI) of 5 and incubated overnight at 37°C. The viral medium was replaced, and 10 μg/mL puromycin was added to purify MSCs stably expressing SeV-TWIST1 (SeV-TW1).

#### FACS sorting of the LRRC15 expressing MSCs in mice and humans

Digested cells or collagenase-treated bone marrow tissues were resuspended in 1%FCS-DMEM supplemented with HEPES (Wako Pure Chemical Corp., Osaka, Japan) and incubated with the following antibodies: anti-LRRC15 antibody (bs-6815R; Bioss Antibodies Inc., Woburn, MA, USA), BV421-conjugated donkey anti-rabbit IgG secondary antibody (Biolegend Japan, Tokyo, Japan), FITC-conjugated anti-CD11b antibody, FITC-conjugated anti-CD45 (30-F11; TONBO Biosciences), and FITC-conjugated anti-TER119. Human MSCs were incubated with anti-human LRRC15 monoclonal antibody (Samrotamab PX-TA1518, Proteogenix, Schiltigheim, France), PE-conjugated anti-human IgG secondary antibody (Thermo Fischer Scientific), FITC-conjugated anti-human CD31, CD45, and TER119.

As a negative control, the cells were treated with APC-, PE-, BV421-, and FITC-conjugated isotype IgGs. CD11b^−^/CD45^-^/Ter119^-^/CD105^+^/Lrrc15^-^/CD11b^−^/CD45^-^/Ter119^-^/CD105^+^/Lrrc15^+^ populations in mice and CD31^-^/CD45^-^/TER119^-^/CD105^+^/LRRC15^-^ or CD31b^−^/CD45^-^/TER119^-^/CD105^+^/LRRC15^+^ populations were sorted using FACS Aria II (BD Biosciences) as MSCs and used for further analysis.

#### Microarray analysis

The microarray gene expression analysis were performed using the Affymetrix Clariom™ S, human (Thermo Fisher Scientific) and the Affymetrix Transcriptome Analysis Console software (Thermo Fisher Scientific).

#### Quantitative RT-PCR (qRT-PCR)

Total RNA was collected from MSCs using TRI Reagent® (Molecular Research Center Inc., Cincinnati, OH, USA) and reverse-transcribed with the PrimeScript® RT Master Mix Kit (TAKARA Bio Inc., Shiga, Japan). rRT-PCR was performed by using Perfect real-time SYBR green II (TAKARA). To prevent amplification of contaminating genomic DNA, we designed all primers to span at least one intron.

#### Western blot (WB) analysis

Cells were homogenized in SDS buffer and centrifuged at 9,000 × *g* for 10 min at 4°C to remove debris. Separation of nuclear fraction and cytoplasmic fraction was performed with NE-PER Nuclear and Cytoplasmic Extraction Reagents (Thermo Fisher Scientific) following manufacture’s instructions. The blotted PVDF membranes were blocked overnight with Block Ace (Dainippon Sumitomo Pharma, Osaka, Japan) and then probed with primary antibodies overnight at 4°C. Detection was performed with horseradish peroxidase (HRP)-conjugated secondary antibodies and Immunostar® LD (Wako) detection reagents.

#### Treatment of primary MSCs with siRNA

Primary MSCs from mice bone marrow tissues were transfected with siRNA against *Twist1* (sense: GAU UUU CAU GGA AAU UAG AdTdT, anti-sense: UCU AAU UUC CAU GAA AAU CdTdT) or scrambled RNA (both are from JBioS Co., LTD., Saitama, Japan) using Lipofectamine® RNAiMAX (Thermo Fisher Scientific) following the manufacturer’s instructions. For the siRNA treatment to the human primary MSCs, a LNA GapmeR antisense oligo nucleotide against *TWIST1* (Hs-TWIST1 common#18, Design ID: 404035-18, Exqon, Vedbaek, Denmark) was used following manufacture’s instructions. As a control for the GapmeR ASO to the *TWIST1* (stated as siTWIST1 in the manuscript) a GampmeR control RNA was used under same condition.

#### Transplantation of the LRRC15^+^ BMMSCs into healthy recipients or BPF model mice

To determine homing properties of the Lrrc15^-^ and Lrrc15^+^ MSCs to the bone marrow, FACS sorted CD11b^-^/CD45^-^/Ter119^-^/CD105^+^/Lrrc15^-^ or CD11b^-^/CD45^-^/Ter119^-^/CD105^+^/Lrrc15^+^ populations were collected from 8-week-old C57BL/6-Tg (CAG-EGFP) male mice (B6-EGFP, Japan SLC, Shizuoka, Japan), and 5×10^4^ cells were transplanted through tail vein of 8-week-old male B6 mice. After 28 days (4 weeks) of cell transplantation, bone marrow tissues were harvested, and EGFP-positive cells were analyzed using FACS Aria II.

To prepare the BPF model, B6 mice were anesthetized using 2% isoflurane. Following anesthesia, pulmonary fibrosis was induced by intratracheal instillation of BLM (2.0 mg/kg, Nippon Kayaku, Japan) in 20 μL phosphate-buffered saline (PBS). Twenty-four hours after BLM administration, the 5×10^4^ CD11b^-^/CD45^-^/Ter119^-^/CD105^+^/Lrrc15^-^ or CD11b^-^/CD45^-^/Ter119^-^/CD105^+^/Lrrc15^+^ cells from B6-EGFP mice were injected via the tail vein. The mice were sacrificed 28 days after MSC transplantation, and the lungs were harvested for qPCR and western blot analysis.

#### Co-expression profiling of TWIST1 and LRRC15 in cancers

We used the cBioPortal online platform to observe co-expression of TWST1 and LRRC15 in various cancers. Analyzed dataset are shown in key resources table.

#### Statistical testing

We performed statistical analysis using JMP 17 software. To test for statistical significance, we used a Student’s *t* test or Tukey’s HSD test. We considered differences statistically significant when p value <0.05. All plots show the mean value per treatment group ±S.D.

## Data Availability

• Microarray and RNAseq data reported in this study is deposited in a public repository with GEO accession numbers GSE228776 and GSE232324. • This paper does not report original code. • Any additional information required to reanalyze the data reported in this paper is available from the lead contact upon request.
