# Supplementary material for: LRRC15 expression indicates high level of stemness regulated by TWIST1 in mesenchymal stem cells
Source: iScience. 2023 May 23;26(7):106946. doi: 10.1016/j.isci.2023.106946 (PMC10391581; doi:10.1016/j.isci.2023.106946)

iScience, Volume *26*

Supplemental information

LRRC15 expression indicates high level of stemness regulated by TWIST1 in mesenchymal stem cells

Kensuke Toriumi, Yuta Onodera, Toshiyuki Takehara, Tatsufumi Mori, Joe Hasei, Kanae Shigi, Natsumi Iwawaki, Toshifumi Ozaki, Masao Akagi, Mahito Nakanishi, and Takeshi Teramura

Figure S1. Quantification of β-galactosidase activity in the senescent MSCs by SPiDER-βGal assay, related to Figure 1.

Left panel shows βGal activity in the mouse MSCs at passage 1 and passage 6. Right panel shows βGal activity in the mouse MSCs at passage 2 and passage 10. Asterisks mean significant difference were detected at P<0.05 (N=3).

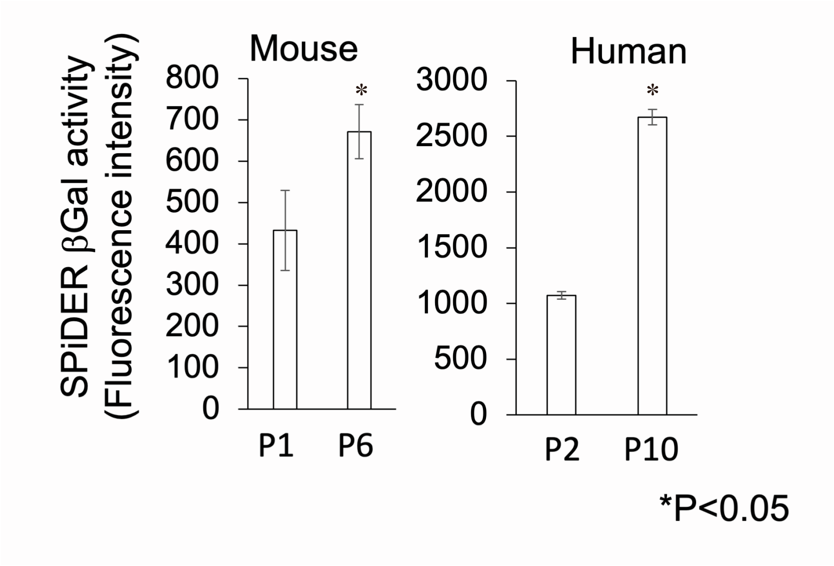

Figure S2. Cell cycle analysis with propidium iodide (PI) for the SeV-GFP transfected control MSCs and the SeV-TWIST1 transfected MSCs, related to Figure 3.

Plot of flow cytometry using PI staining for cell cycle analysis of MSCs transfected with SeV-GFP (left) and SeV-TWIST1 (right).

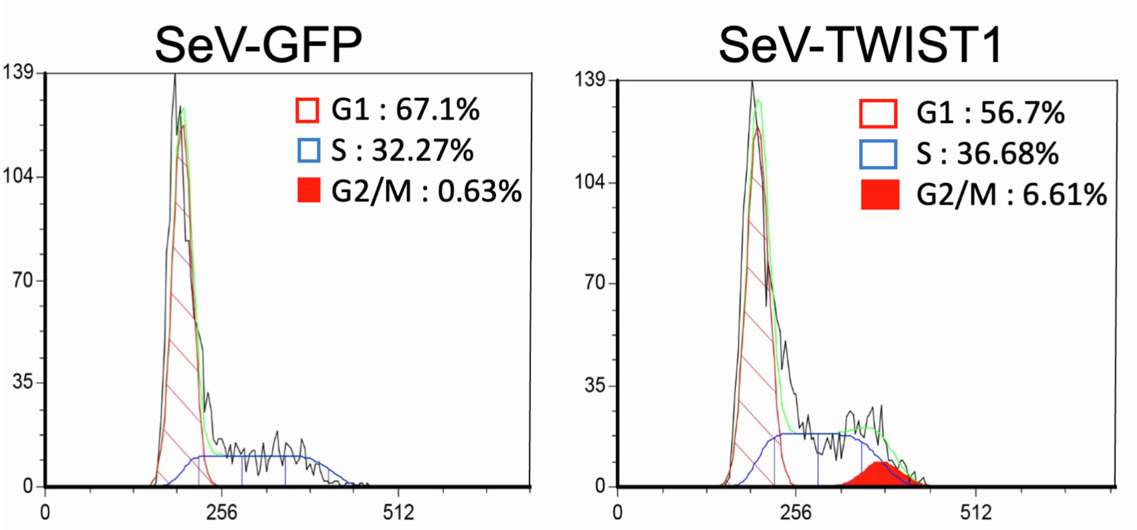

Figure S3. Specificity of CD29 as a human MSC marker for cell sorting, related to Figure 6.

More than 90% of primary human BMMSCs co-expressed CD90 and CD29.

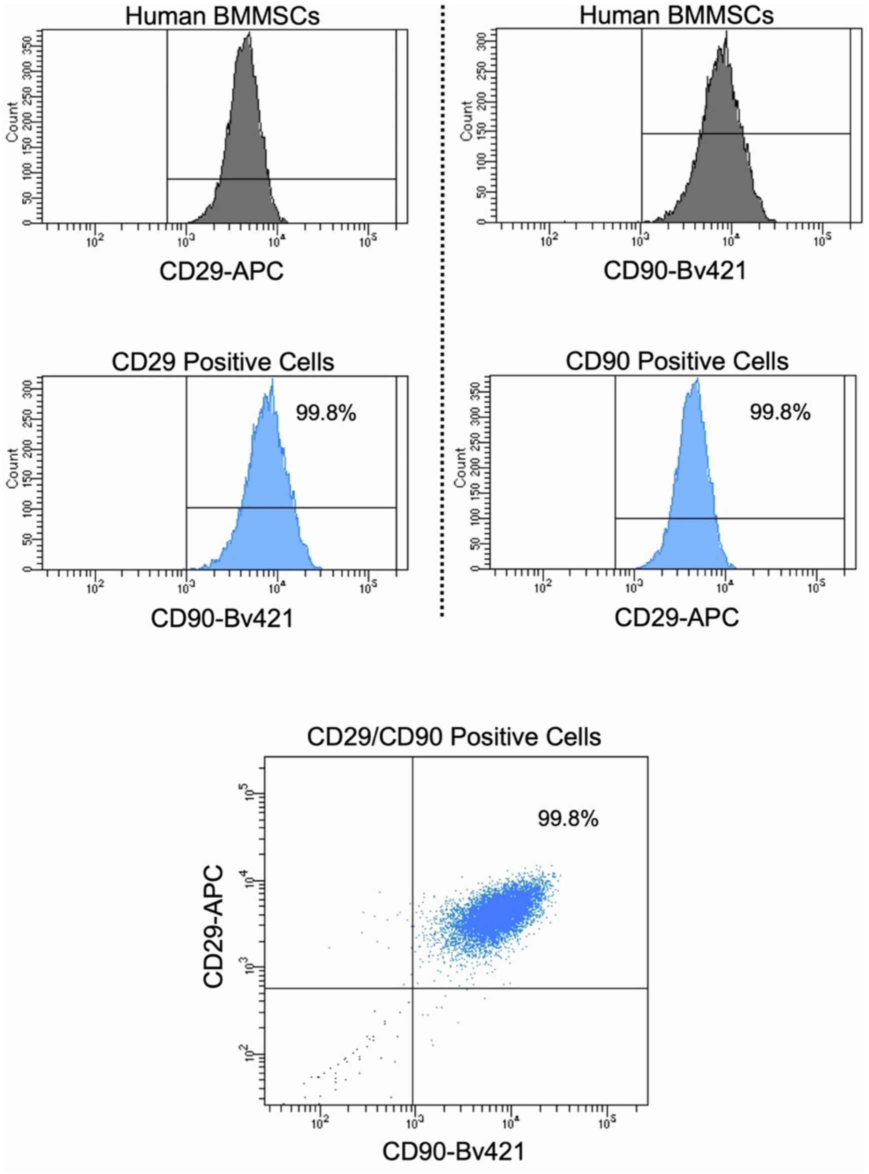

Figure S4. Expression of cell surface MSC markers on the LRRC15⁺ cells in the primary cultures of human MSCs, related to Figure 6.

FACS analysis for CD29 (upper left), CD73 (upper right), CD90 (lower left), and CD105 (lower right).

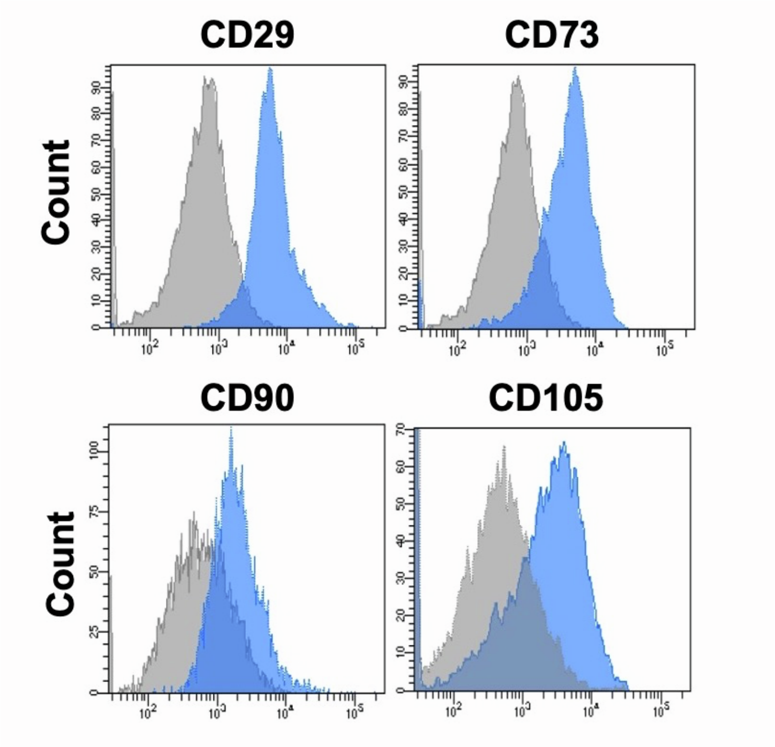

Figure S5. Histological analysis of the control and the LRRC15^{+} MSC-transplanted BLM model mice, related to Figure 7.

Histological analysis with Masson's trichrome staining detects collagen deposition in the lung (green). Arrow heads show collagen accumulation.

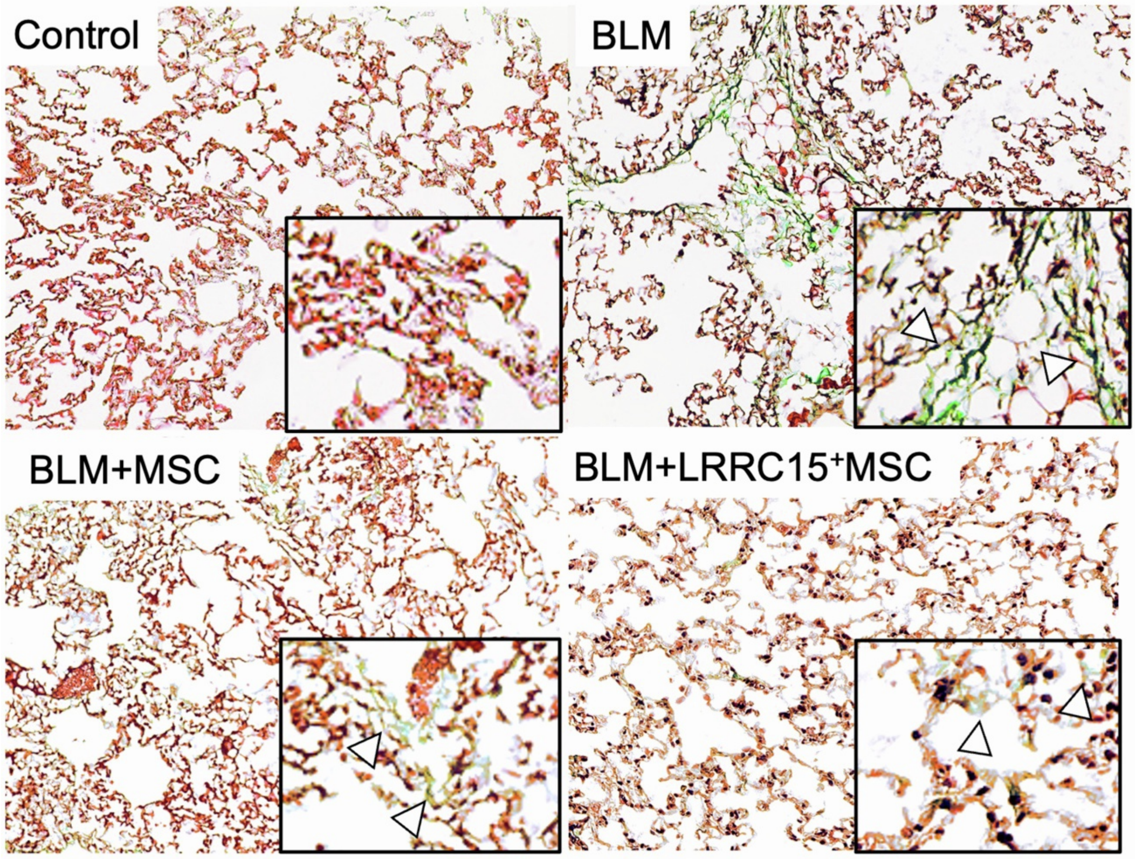

Figure S6. Expression changes of the EMT-related genes including endogenous TWIST1 in the MSCs transfected with SeV-TWIST1 or siRNA against TWIST1 (siTWIST1), related to Figure 3.

A, EMT-related gene expression in the no treatment control MSCs (NTC), the control MSCs transfected with SeV-GFP, and TWIST1 overexpressing MSCs (SeV-TWIST1). B, EMT-related gene expression in the no treatment control MSCs (NTC), the control MSCs transfected with scrambled sequence RNA, and TWIST1 targeted siRNA-transfected MSCs (siTWIST1).

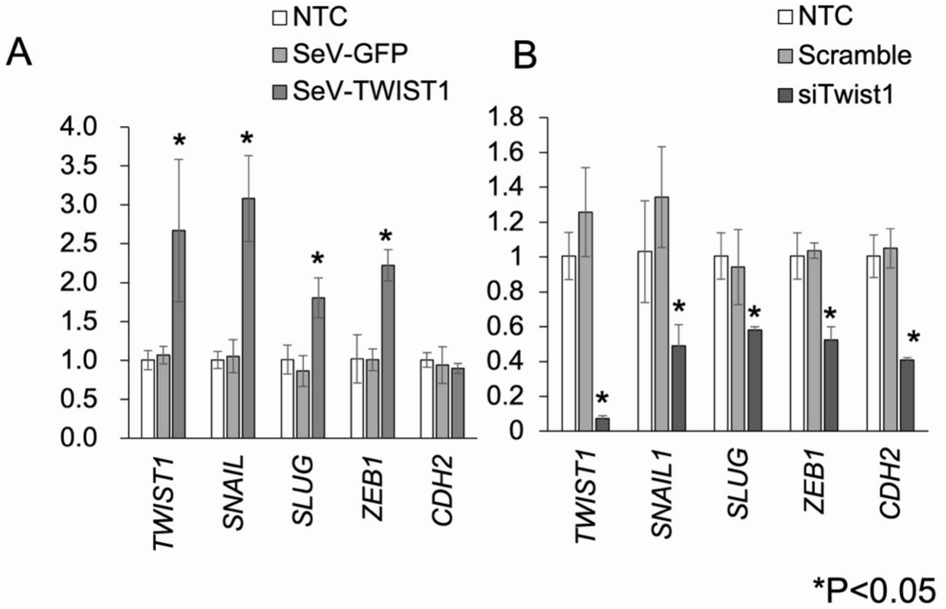

Figure S7. Effect of TWSIT1 overexpression on in vitro differentiation potentials of human MSCs, related to Figure 3.

A, Gene expressions of adipocyte, chondrocyte, and osteoblast markers were supressed in the TWIST1-overexpressing MSCs (TW1) when observed on day14 after in vitro differentiation induction.

B, Alizalin red staining for detection of calcification by osteoblast differentiation, alucian blue staining showing cartilage matrix deposition by chondrocyte differentiation, and oil-red o staining for detection of lipid accumulation by adipocyte differentiation. All were inhibited by the TWIST1 expression.

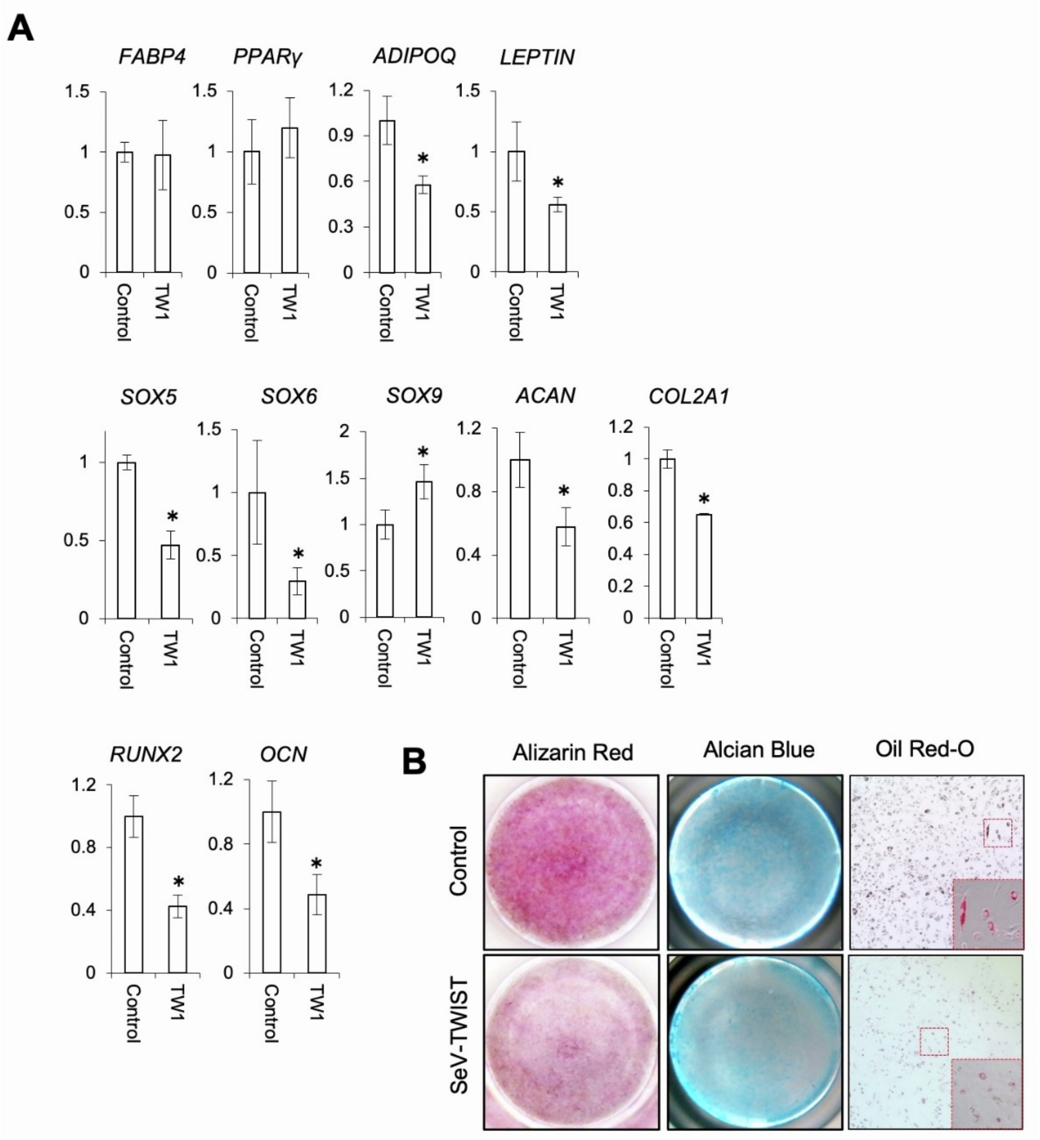

Figure S8. Positive gene expression correlations between TWIST1 (x-axis) and LRRC15 (y-axis) in various cancer types, related to Figure 4 and STAR Methods. TWIST1 and LRRC15 expression in bladder cancers, rectal cancer, colorectal adenocarcinoma, esophageal adenocarcinoma, and lung adenocarcinoma were analyzed based on The Cancer Genome Atlas (TCGA).

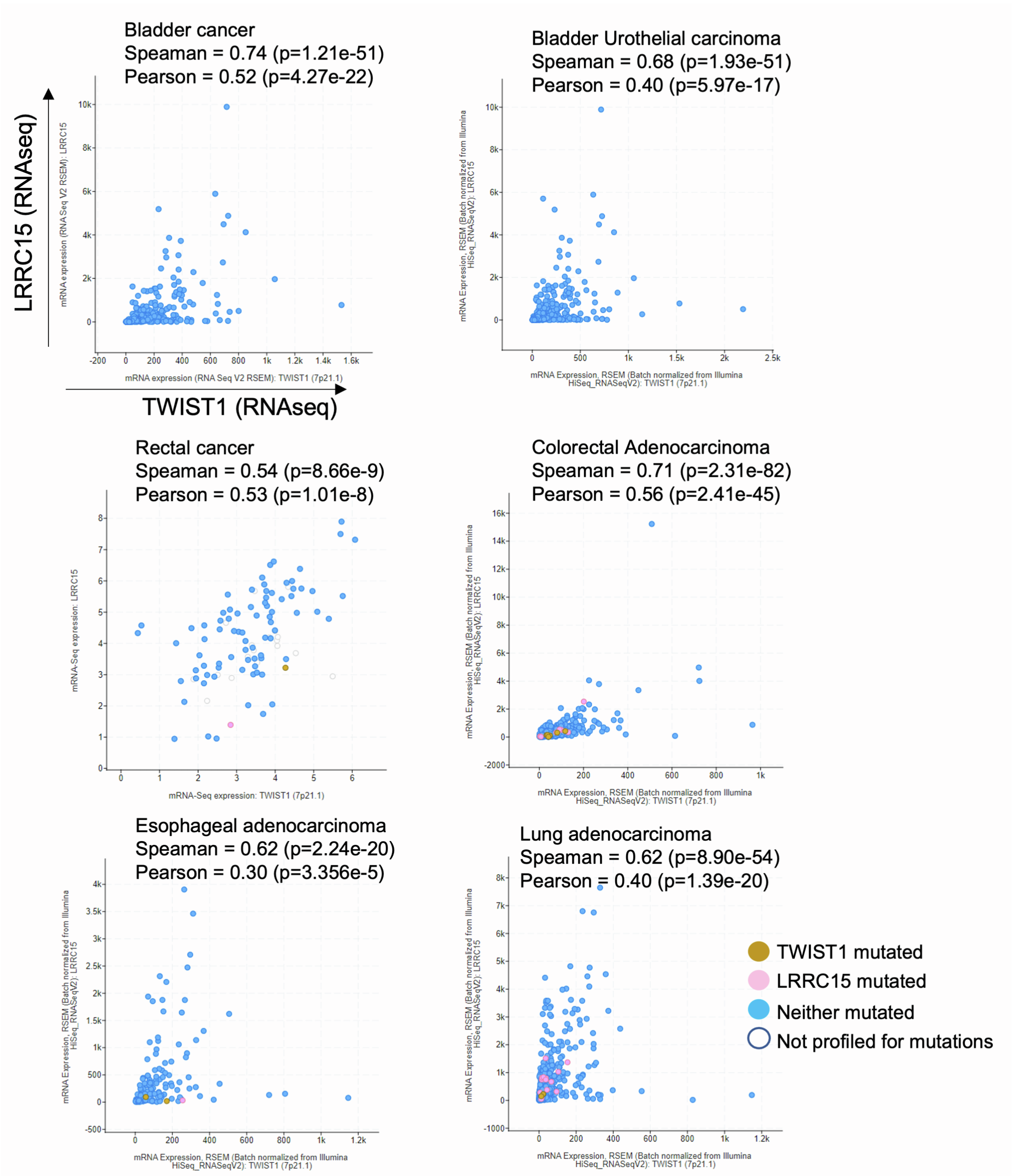

Figure S9. Positive gene expression correlations between TWIST1 (x-axis) and LRRC15 (y-axis) in various cancer types, related to Figure 4 and STAR Methods. TWIST1 and LRRC15 expression in pancreatic cancers, mesothelioma, sarcoma, thyroid carcinoma, and kidney renal clear cell carcinoma were analyzed based on The Cancer Genome Atlas (TCGA).

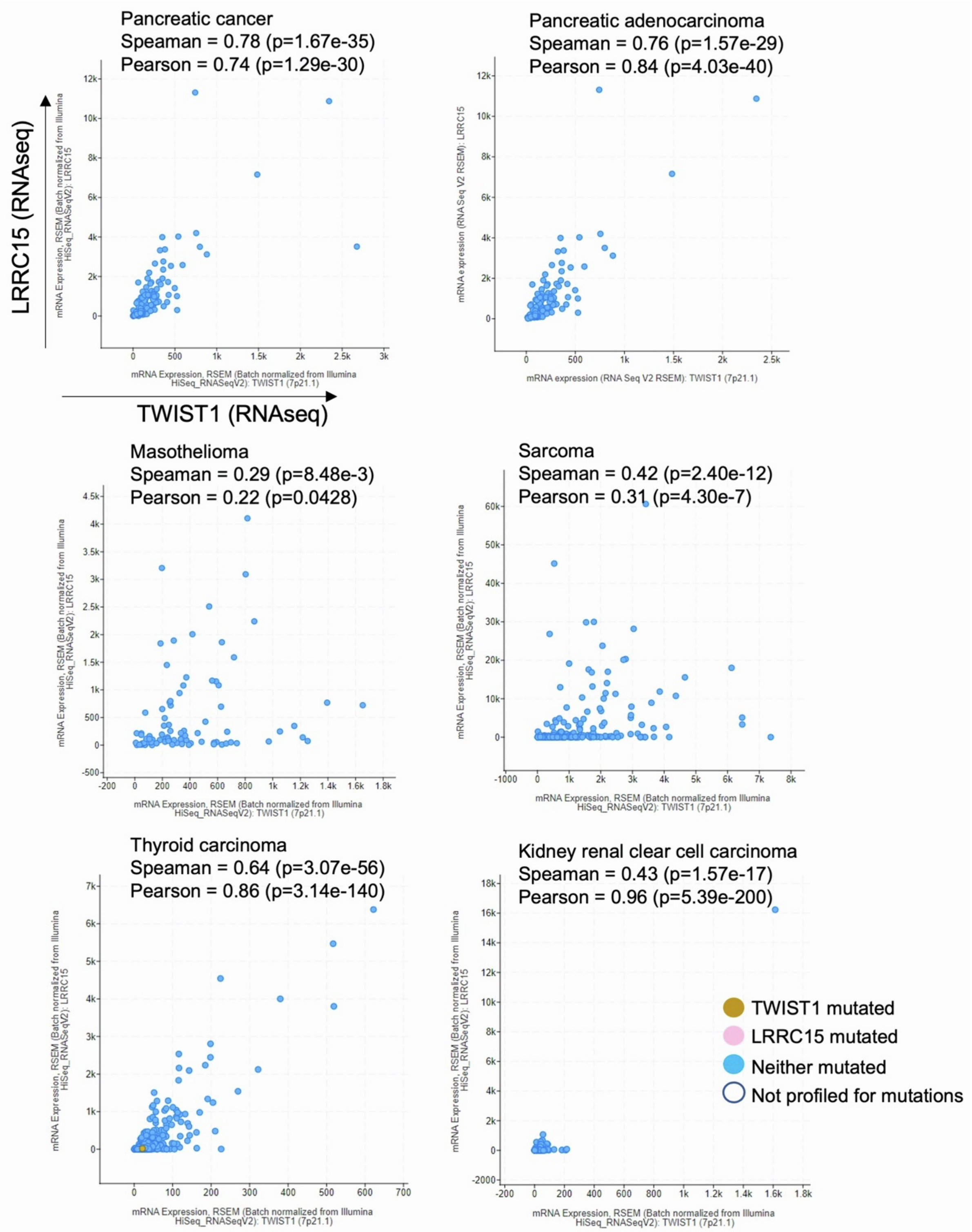

Figure S10. RNA sequence-based analysis of the nascent RNA by NET-CAGE (Native Elongating Transcript Cap Analysis of Gene Expression) in the SeV-TWIST1 transfected HDF cells, related to Figure 4.

The NET-CAGE analysis showed changes of transfection activity of each gene accurately. A, dot plot comparing gene expression differences in the SeV-GFP-transfected HDFs and the SeV-TWIST1-transfected HDFs.

B, list of the stemness-related genes including LRRC15 and changes of transcriptional activity by TWIST1 introduction. This result shows that LRRC15 expression was activated by TWIST1 (its fold-change was highlighted by yellow).

A

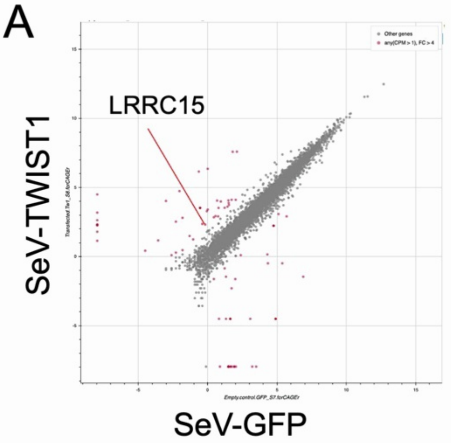

B

	Base mean	Fold Change	lfcSE	p-value
Endogenous TWIST1	898.41	0.925	0.423	0.029
LRRC15	72.832	2.452	0.996	0.014
ID1	1668.667	1.672	1.507	0.267
ID2	660.476	1.182	1.118	0.29
BMI1	451.624	-0.492	0.372	0.186
Zeb1	1687.408	0.086	0.256	0.736
NOTCH1	516.897	-0.13	0.316	0.681
FOXP1	5655.795	0.039	0.233	0.868
ETV1	475.394	0.128	0.323	0.691

Figure S11. Effect of siLRRC15 treatment in the human MSCs, related to Figure 6. Expression changes of TWIST1 and cell proliferation-related genes by suppression of LRRC15 genes in the human MSCs. Asterisks mean significant difference were detected at $P<0.05$ (N=3).

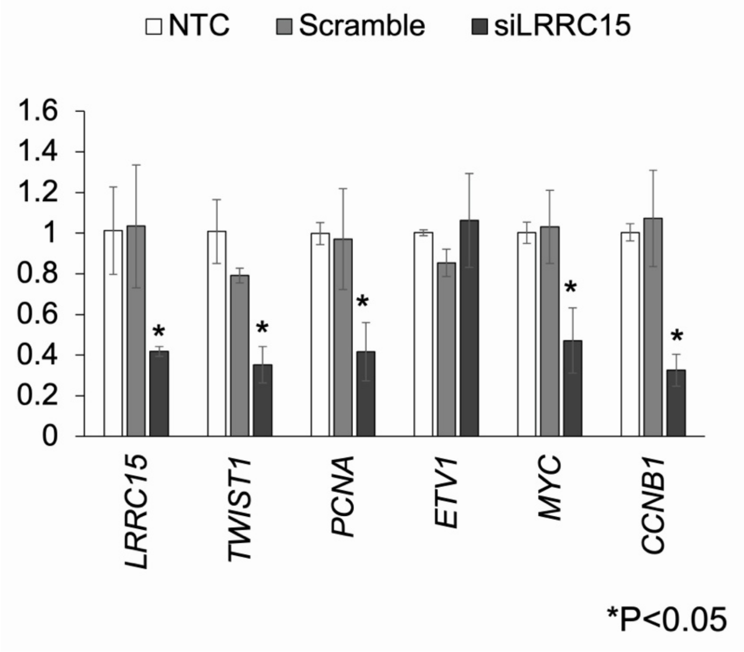

Supplement: Document S1. Figures S1–S11 [file mmc1.pdf]
